# Diagnosing Solid Lesions in the Pancreas With Multimodal Artificial Intelligence: A Randomized Crossover Trial

**DOI:** 10.1001/jamanetworkopen.2024.22454

**Published:** 2024-07-19

**Authors:** Haochen Cui, Yuchong Zhao, Si Xiong, Yunlu Feng, Peng Li, Ying Lv, Qian Chen, Ronghua Wang, Pengtao Xie, Zhenlong Luo, Sideng Cheng, Wujun Wang, Xing Li, Dingkun Xiong, Xinyuan Cao, Shuya Bai, Aiming Yang, Bin Cheng

**Affiliations:** 1 Department of Gastroenterology and Hepatology, Tongji Hospital, Tongji Medical College, Huazhong University of Science and Technology, Wuhan, China; 2 Department of Gastroenterology, Peking Union Medical College Hospital, Chinese Academy of Medical Sciences & Peking Union Medical College, Beijing, China; 3 Department of Gastroenterology, Beijing Friendship Hospital, Capital Medical University, Beijing, China; 4 Department of Gastroenterology, Nanjing Drum Tower Hospital, Affiliated Drum Tower Hospital, Medical School of Nanjing University, Nanjing, Jiangsu, China; 5 Department of Surgery, University of Pittsburgh School of Medicine, Pittsburgh, Pennsylvania; 6 Department of Electrical and Computer Engineering, University of California San Diego, La Jolla; 7 Department of Computer Science, Algoma University, Sault Ste. Marie, Ontario, Canada; 8 Wuhan EndoAngel Medical Technology Company, Wuhan, China

## Abstract

**Importance:**

Diagnosing solid lesions in the pancreas via endoscopic ultrasonographic (EUS) images is challenging. Artificial intelligence (AI) has the potential to help with such diagnosis, but existing AI models focus solely on a single modality.

**Objective:**

To advance the clinical diagnosis of solid lesions in the pancreas through developing a multimodal AI model integrating both clinical information and EUS images.

**Design, Setting, and Participants:**

In this randomized crossover trial conducted from January 1 to June 30, 2023, from 4 centers across China, 12 endoscopists of varying levels of expertise were randomly assigned to diagnose solid lesions in the pancreas with or without AI assistance. Endoscopic ultrasonographic images and clinical information of 439 patients from 1 institution who had solid lesions in the pancreas between January 1, 2014, and December 31, 2022, were collected to train and validate the joint-AI model, while 189 patients from 3 external institutions were used to evaluate the robustness and generalizability of the model.

**Intervention:**

Conventional or AI-assisted diagnosis of solid lesions in the pancreas.

**Main Outcomes and Measures:**

In the retrospective dataset, the performance of the joint-AI model was evaluated internally and externally. In the prospective dataset, diagnostic performance of the endoscopists with or without the AI assistance was compared.

**Results:**

The retrospective dataset included 628 patients (400 men [63.7%]; mean [SD] age, 57.7 [27.4] years) who underwent EUS procedures. A total of 130 patients (81 men [62.3%]; mean [SD] age, 58.4 [11.7] years) were prospectively recruited for the crossover trial. The area under the curve of the joint-AI model ranged from 0.996 (95% CI, 0.993-0.998) in the internal test dataset to 0.955 (95% CI, 0.940-0.968), 0.924 (95% CI, 0.888-0.955), and 0.976 (95% CI, 0.942-0.995) in the 3 external test datasets, respectively. The diagnostic accuracy of novice endoscopists was significantly enhanced with AI assistance (0.69 [95% CI, 0.61-0.76] vs 0.90 [95% CI, 0.83-0.94]; *P* < .001), and the supplementary interpretability information alleviated the skepticism of the experienced endoscopists.

**Conclusions and Relevance:**

In this randomized crossover trial of diagnosing solid lesions in the pancreas with or without AI assistance, the joint-AI model demonstrated positive human-AI interaction, which suggested its potential to facilitate a clinical diagnosis. Nevertheless, future randomized clinical trials are warranted.

**Trial Registration:**

ClinicalTrials.gov Identifier: NCT05476978

## Key Points

**Question:**

Can a multimodal artificial intelligence (AI) model facilitate a clinical diagnosis of solid lesions in the pancreas?

**Findings:**

In this randomized crossover trial based on a prospective dataset of 130 patients who underwent endoscopic ultrasonographic (EUS) procedures, a multimodal AI model, incorporating endoscopic EUS images and clinical data, demonstrated robustness across internal and external cohorts (the area under the curve of the joint-AI model ranged from 0.996 in the internal test dataset to 0.955, 0.924, and 0.976 in the 3 external test datasets, respectively). In addition, the performance of novice endoscopists was significantly enhanced with AI assistance.

**Meaning:**

This study suggests that endoscopists of varying expertise can efficiently cooperate with this multimodal AI model, establishing a proof-of-concept study for human-AI interaction in the management of solid lesions in the pancreas.

## Teaser

This randomized crossover trial discusses the development of a multimodal artificial intelligence (AI) model integrating both clinical information and endoscopic ultrasonographic images for the diagnosis of solid lesions in the pancreas.

## Introduction

Pancreatic cancer is a prevalent cause of masses in the pancreas, with an overall 5-year survival rate of approximately 10%.^1^ Endoscopic ultrasonography (EUS) has emerged as a valuable technique in diagnosing pancreatic cancer, showing superior sensitivity to computed tomography and magnetic resonance imaging, particularly for tumors less than 3 cm in diameter.^2^ However, other less-malignant neoplasms (eg, a pancreatic neuroendocrine tumor or a solid pseudopapillary tumor) and benign pancreatic conditions (eg, chronic pancreatitis and autoimmune pancreatitis) can also manifest as masses in the pancreas. Because the management and prognosis of pancreatic cancer is vastly different from that of other lesions, a precise diagnosis is paramount.^3,4,5^ Nevertheless, the specificity of EUS in discriminating malignant neoplasms from benign masses is suboptimal, ranging from 50% to 60%.^6^ Although EUS-guided, fine-needle aspiration or biopsy (EUS-FNA/B) has significantly improved overall diagnostic accuracy, achieving a reliable level of 80% to 90% and a specificity of 92% to 99%, concerns linger about the relatively low and unstable sensitivity and negative predictive value (NPV) of the technique, falling between 80% to 89% for sensitivity and 46% to 75% for NPV.^7,8,9,10^ This finding highlights the need for complementary techniques to provide additional information.

In recent years, artificial intelligence (AI) models have shown potential in oncology, contributing to screening, diagnosis, treatment guidance, and prognosis prediction.^11^ For medical image analysis, convolutional neural networks (CNNs) are one of the most widely used deep learning algorithms. Although CNN models had shown promise in differentiating pancreatic cancer in EUS images, prior studies predominantly lacked external validation, impeding the pathway to clinical translation.^12,13,14,15,16,17^ Furthermore, the existing deep learning models operate on only a single modality, overlooking the potential diagnostic effects of other aspects, such as medical history, laboratory tests, and radiology results. Integrating multiple modalities is likely to improve the robustness of the diagnostic model.^18,19,20^ In addition, multimodal AI models are expected to be more applicable than models using a single modality to clinical practice.^21^ As in clinical practice, physicians make diagnoses based on a comprehensive assessment of all the information available.

In this study, we developed a multimodal AI model, using both EUS images and clinical information to distinguish carcinoma from noncancerous lesions, and tested this model in internal, external, and prospective datasets. We also evaluated the assisting potential of the model in a crossover trial and examined whether interpretability analyses could facilitate clinical application.

## Methods

### Study Design and Participants

The primary objective of this study was to develop and validate a multimodal AI model capable of differentiating carcinoma from noncancerous lesions in the pancreas. Endoscopic ultrasonographic images and clinical information were retrospectively collected between January 1, 2014, and December 31, 2022, from 4 institutions across China. The study was approved by the ethical committee of Tongji Hospital, Tongji Medical College, Huazhong University of Science and Technology and registered at ClinicalTrial.gov (NCT05476978; trial protocol in Supplement 1). For retrospective datasets used in the development and testing of the multimodal AI model, informed consent was waived because only preexisting clinical data were collected. Regarding the prospective dataset in the crossover study, all patients provided written informed consent for the study participation. The randomized crossover trial followed the Consolidated Standards of Reporting Trials (CONSORT) reporting guideline.

Patients (aged ≥18 years) with solid lesions in the pancreas were included. The carcinoma lesions included pancreatic ductal adenocarcinoma, acinar cell carcinoma, and squamous cell carcinoma in the pancreas. The noncancerous lesions comprised pancreatic neuroendocrine tumors, solid pseudopapillary tumors, autoimmune pancreatitis, chronic pancreatitis, and tuberculosis. Pancreatic ductal adenocarcinoma, acinar cell carcinoma, squamous cell carcinoma, pancreatic neuroendocrine tumors, and solid pseudopapillary tumors were diagnosed pathologically by specimens obtained from EUS-FNA/B or surgery. Autoimmune pancreatitis was diagnosed according to the International Consensus Diagnostic Criteria for autoimmune pancreatitis.^22^ Chronic pancreatitis was diagnosed if there were neither malignant neoplasms detected in specimens acquired from EUS-FNA/B and/or surgery nor a rapid progression of diseases in the pancreas observed during the 6-month follow-up period. The diagnosis of tuberculosis was based on a consensus reached through pathologic findings, GeneXpert analysis, and the response to the antituberculosis treatment.

Data derived from Wuhan Tongji Hospital (WHTJH) were used as a training dataset and an internal testing dataset. External testing datasets were obtained from Nanjing Drum Tower Hospital (NJDTH), Peking Union Medical College Hospital (PUMCH), and Beijing Friendship Hospital (BJFH) (eMethods and eFigure 1 in Supplement 2).

### Development of AI Models

To integrate information from both EUS images and clinical data, a CNN (model 1) was trained on EUS images using a ResNet-50 architecture (Microsoft Visio) with transfer learning, and machine learning models (model 2) were trained on 36 clinical features from 5 categories. Model 3, a multilayer perceptron model with 2 fully connected layers, used 3 data fusion strategies (A, B, and C) to combine features, probabilities, or predictions from model 1 and model 2. The final output of the 3 models was binary, being either carcinoma or noncarcinoma.

During the development of model 2, the optimal combination of algorithm and features for each category was determined based on diagnostic accuracy. A random forest was used for personal history and radiology findings, while a decision tree was used for medical history. A support vector machine was used for clinical symptoms, and a gradient-boosting decision tree was applied to laboratory tests.

The training of model 1 and model 3 was performed using Adam optimizers, with an initial learning rate of 0.001 and a batch size of 16. Overfitting was mitigated using a dropout rate of 0.5 and early stopping, which halted training if validation loss did not decrease for 10 epochs. The actual training epochs were 64 for model 1 and 24 for model 3.

Interpretability analyses, including gradient-weighted class activation mapping (Grad-CAM) and Shapley Additive Explanation (SHAP) algorithms, were implemented. Detailed information about the training process is provided in the eMethods in Supplement 2.

### Procedures of the Crossover Trial

After the completion of the training process, consecutive patients who underwent EUS examinations and received a definitive diagnosis of lesions in the pancreas were prospectively enrolled at WHTJH and PUMCH from January 1 to June 30, 2023. The clinical information and EUS images were collected and preprocessed.

**Figure 1.**
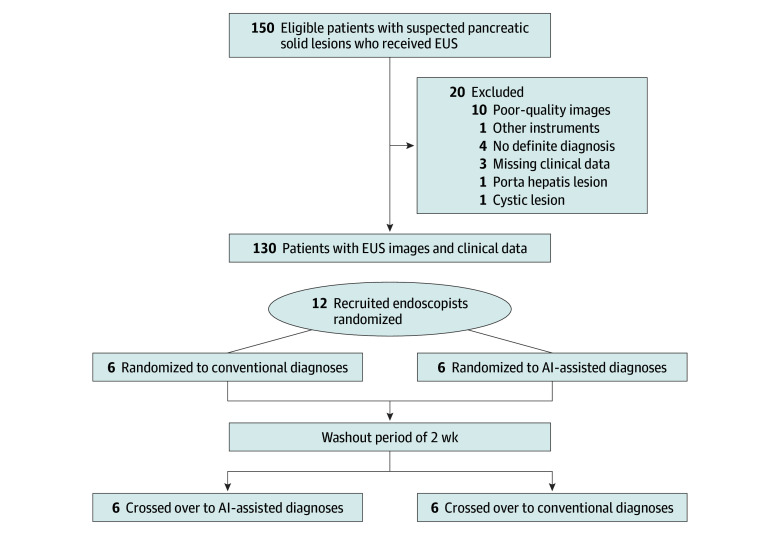
Flow Diagram for the Crossover Trial AI indicates artificial intelligence; and EUS, endoscopic ultrasonography.

A crossover trial was conducted, in which endoscopists were required to diagnose the lesions in the pancreas based on clinical information and EUS images, with or without the predictions provided by AI models (Figure 1). To help endoscopists better understand the nature of the models, model 1 was named the EUS-CNN model, and model 3 was named the joint-AI model during the crossover trial.

Twelve endoscopists from 9 centers across China were recruited, including 2 experts (who annually performed ≥300 EUS procedures with >10 years of experience), 4 senior endoscopists (who annually performed ≥150 EUS procedures with >5 years of experience), and 6 novice endoscopists (with >1 year of experience in EUS). None of the endoscopists participated in the data collection and preprocessing, and they were all masked to the personal information, EUS reports, pathologic results, and clinical diagnosis of the involved patients. According to their level of expertise, endoscopists were randomly assigned in a 1:1 ratio by an independent researcher (Y.Z.), who was blinded to the endoscopists’ personal information, to either the group that initially diagnosed with AI assistance or the group that diagnosed without AI assistance. After a washout period of 2 weeks, the 2 groups switched.

After completion of the study, results of interpretability analyses were additionally provided to expert and senior endoscopists. A questionnaire was sent to endoscopists after the crossover trial (eFigure 2 in Supplement 2).

### Statistical Analysis

The performance of model 1, model 3, and endoscopists were evaluated by metrics including accuracy, sensitivity, specificity, positive predictive value, NPV, and area under the curve (AUC). This evaluation was conducted in 2 phases: the image phase and the patient phase. In the image phase, the CNN models’ outputs for each individual image were extracted, and the diagnostic performance was evaluated. In the patient phase, we used a threshold of 3 positive images for the diagnosis of carcinoma. The optimal cutoff value of the receiver operating characteristic curve was determined when the Youden index was maximized. The McNemar test was used to compare the differences in accuracy, sensitivity, and specificity. Generalized score statistics were used to compare the positive predictive value and NPV. A Wilcoxon matched-pairs signed rank test was used to compare the effect of models on the diagnosis of endoscopists. A χ^2^ analysis was used to compare the rejection rate and the endoscopists’ preferences to the models. All data and statistical analyses were performed using SPSS, version 26.0 (IBM SPSS Statistics) and R, version 4.2.1 (R Project for Statistical Computing). A 2-sided *P* < .05 was considered statistically significant.

## Results

Data from a total of 789 patients who underwent EUS procedures for solid lesions in the pancreas were retrospectively collected between January 1, 2014, and December 31, 2022, from 4 centers across China. After excluding patients due to poor-quality images (n = 65) and use of different EUS instruments (n = 96), 628 patients were included (400 male participants [63.7%] and 228 female participants [36.3%]; mean [SD] age, 57.7 [27.4] years). Of the 628 patients included, data from 439 patients (69.9%) were collected at our center (WHTJH); these patients were randomly assigned to the training and validation dataset (351 cases with 6181 EUS images) and the internal testing dataset (88 cases with 1545 EUS images), with a ratio of 8:2 (eTable 1 in Supplement 2). The external testing datasets collected from NJDTH, PUMCH, and BJFH included 189 patients (30.1%) with 1205 still images. After the training process, 130 patients (81 male participants [62.3%] and 49 female participants [37.7%]; mean [SD] age, 58.4 [11.7] years) were prospectively recruited from January 1 to June 30, 2023, from WHTJH and PUMCH. Detailed information of the included patients is provided in eTable 1 in Supplement 2.

**Figure 2.**
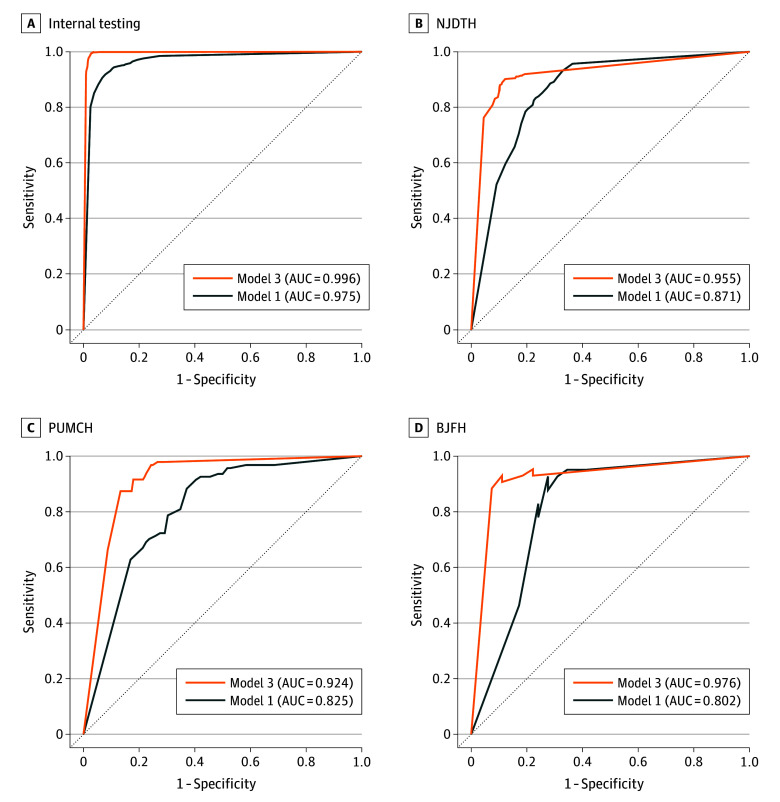
Performance of the Artificial Intelligence Models in Differentiating Carcinoma and Noncancerous Lesions AUC indicates area under the curve; BJFH, Beijing Friendship Hospital; NJDTH, Nanjing Drum Tower Hospital; and PUMCH, Peking Union Medical College Hospital.

The diagnostic performance of model 1 is shown in eTable 2 in Supplement 2. For internal testing, it achieved an AUC of 0.975 (95% CI, 0.969-0.981) in the image phase. However, model 1 had lower AUCs in the external testing datasets, between 0.802 (95% CI, 0.648-0.941) and 0.871 (95% CI, 0.848-0.892) (Figure 2).

We aimed to enhance the generalizability of the model by incorporating relevant clinical information. Several machine learning algorithms were used to select significant clinical features from different categories, and 24 features were extracted from the original 36 features according to their diagnostic accuracy (eTable 3 in Supplement 2). During the development of model 3, the diagnostic performance of the 3 data fusion strategies was compared. In the image phase, strategy B achieved the highest AUC of 0.996 (95% CI, 0.993-0.998) (eFigure 3 in Supplement 2), with an accuracy of 0.98 (95% CI, 0.98-0.99) (eTable 4 in Supplement 2). Similarly, strategy B achieved the highest AUC in the patient phase (eFigure 3 in Supplement 2).

Model 3 built on strategy B was further evaluated on external testing datasets. It attained an accuracy of 0.84 (95% CI, 0.79-0.87) to 0.89 (95% CI, 0.87-0.91) in the image phase and an accuracy of 0.84 (95% CI, 0.74-0.91) to 0.91 (95% CI, 0.73-0.98) in the patient phase (Table 1). Model 3 demonstrated enhanced performance compared with the single-modal model 1. In the image phase, the AUC of model 3 showed a significant increase compared with model 1 in NJDTH (0.955 [95% CI, 0.940-0.968] vs 0.871 [95% CI, 0.848-0.892]; *P* < .001), PUMCH (0.924 [95% CI, 0.888-0.955] vs 0.825 [95% CI, 0.783-0.868]; *P* < .001), and BJFH (0.976 [95% CI, 0.942-0.995] vs 0.802 [95% CI, 0.648-0.941]; *P* < .001) (Figure 2). Similarly, model 3 outperformed model 1 in the patient phase (eFigure 4 in Supplement 2). A representative user interface of model 3 is demonstrated in the Video.

**Table 1.** Diagnostic Performance of Model 3 in Internal and External Test Datasets

Datasets	Sensitivity (95% CI)	Specificity (95% CI)	PPV (95% CI)	NPV (95% CI)	Accuracy (95% CI)
Image phase					
Internal testing	0.99 (0.98-0.99)	0.98 (0.96-0.98)	0.98 (0.97-0.99)	0.99 (0.98-0.99)	0.98 (0.98-0.99)
NJDTH	0.88 (0.84-0.91)	0.89 (0.86-0.91)	0.83 (0.79-0.87)	0.92 (0.90-0.94)	0.89 (0.87-0.91)
PUMCH	0.96 (0.90-0.98)	0.77 (0.70-0.82)	0.69 (0.61-0.77)	0.97 (0.93-0.99)	0.84 (0.79-0.87)
BJFH	0.93 (0.81-0.98)	0.78 (0.59-0.89)	0.87 (0.74-0.94)	0.88 (0.69-0.96)	0.87 (0.77-0.93)
Patient phase					
Internal testing	0.99 (0.97-1.00)	0.98 (0.94-0.99)	0.98 (0.96-0.99)	0.98 (0.95-0.99)	0.98 (0.97-0.99)
NJDTH	0.92 (0.82-0.97)	0.88 (0.77-0.94)	0.89 (0.77-0.95)	0.92 (0.81-0.97)	0.90 (0.83-0.94)
PUMCH	0.92 (0.77-0.98)	0.78 (0.63-0.89)	0.76 (0.59-0.87)	0.94 (0.79-0.98)	0.84 (0.74-0.91)
BJFH	0.93 (0.70-0.99)	0.88 (0.53-0.98)	0.93 (0.70-0.99)	0.88 (0.53-0.98)	0.91 (0.73-0.98)

Abbreviations: BJFH, Beijing Friendship Hospital; NJDTH, Nanjing Drum Tower Hospital; NPV, negative predictive value; PPV, positive predictive value; PUMCH, Peking Union Medical College Hospital.

**Video.** User Interface of the Joint Artificial Intelligence (AI) Model This video demonstrates the operating interface of the joint AI model (model 3). During the endoscopic ultrasonography procedure, a red square dynamically indicates the lesion detected by the model. Model 1 generates real-time predictions, and the gradient-weighted class activation mapping creates heatmaps based on images captured by endoscopists. Toward the end of the procedure, the joint AI model provides the final diagnosis according to both the captured images and clinical data. Shortly after the joint AI model delivers its prediction, the Shapley additive explanations algorithm calculates the impact of each category on the output of the joint AI model. In this video, the patients eventually received pathological diagnoses of pancreatic cancer and solid pseudopapillary tumor, respectively.

**Figure 3.**
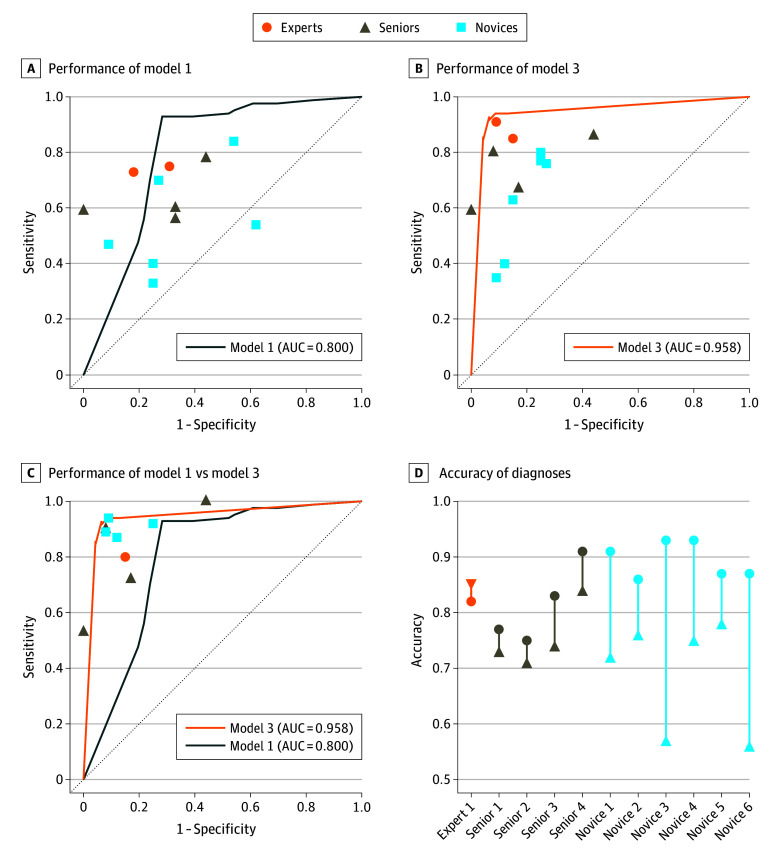
Diagnostic Performance of Endoscopists and Artificial Intelligence Models in the Crossover Trial AUC indicates area under the curve.

A prospective crossover trial was conducted to further evaluate the performance and assisting ability of the AI models (eTable 5 in Supplement 2). When solely EUS images were provided, model 1 outperformed the endoscopists (Figure 3A), which was more sensitive (0.93 [95% CI, 0.85-0.97]) than expert endoscopists (0.74 [95% CI, 0.59-0.85]; *P* = .02), senior endoscopists (0.62 [95% CI, 0.50-0.73]; *P* < .001), and novice endoscopists (0.56 [95% CI, 0.46-0.66]; *P* < .001) (eTable 6 in Supplement 2). When diagnoses were based on both clinical information and EUS images, model 3 remained superior in diagnostic performance (Figure 3B). Model 3 was more sensitive than senior endoscopists (0.92 [95% CI, 0.84-0.96] vs 0.72 [95% CI, 0.61-0.82]; *P* = .002) and more accurate than senior endoscopists (0.92 [95% CI, 0.86-0.96] vs 0.77 [95% CI, 0.68-0.84]; *P* = .001); it was also more sensitive than novice endoscopists (0.61 [95% CI, 0.51-0.70]; *P* < .001) and more accurate than novice endoscopists (0.69 [95% CI, 0.61-0.76]; *P* < .001) (eTable 7 in Supplement 2). With additional assistance from AI (Figure 3C and D), novice endoscopists demonstrated a significant improvement in sensitivity (0.91 [95% CI, 0.83-0.95]; *P* < .001) and accuracy (0.90 [95% CI, 0.83-0.94]; *P* < .001). However, expert and senior endoscopists did not benefit from AI assistance (Table 2). The total rejection rates of the expert and senior endoscopists were significantly higher than that of novice endoscopists (odds ratio, 2.15 [95% CI, 1.12-4.16]; *P* = .02) (eTable 8 in Supplement 2). Through supplementing results of interpretability analyses (eFigures 5 and 6 in Supplement 2), the total rejection rates of the expert and senior endoscopists reached a level comparable to that of the novice endoscopists (odds ratio, 0.71 [95% CI, 0.32-1.58]; *P* = .40) (eTable 8 in Supplement 2). Correspondingly, a decrease in the false rejection rate was also observed.

**Table 2.** Comparing Performance of Endoscopists With or Without AI Assistance

Metrics	Expert endoscopists (n = 2)				Senior endoscopists (n = 4)				Novice endoscopists (n = 6)			
	Without AI	With AI^a^	*P* value	Without AI		With AI	*P* value	Without AI		With AI	*P* value
Sensitivity (95% CI)	0.88 (0.75-0.95)	0.80 (0.58-0.92)	>.99	0.72 (0.61-0.82)		0.78 (0.67-0.86)	.39	0.61 (0.51-0.70)		0.91 (0.83-0.95)	<.001
Specificity (95% CI)	0.88 (0.69-0.96)	0.85 (0.58-0.96)	>.99	0.84 (0.71-0.92)		0.84 (0.71-0.92)	>.99	0.81 (0.70-0.89)		0.88 (0.77-0.94)	.34
PPV (95% CI)	0.92 (0.80-0.97)	0.89 (0.67-0.97)	.66	0.88 (0.77-0.94)		0.88 (0.78-0.94)	.89	0.84 (0.74-0.91)		0.92 (0.85-0.96)	.10
NPV (95% CI)	0.80 (0.62-0.91)	0.73 (0.48-0.89)	.58	0.66 (0.53-0.77)		0.71 (0.58-0.82)	.57	0.56 (0.46-0.66)		0.85 (0.74-0.92)	<.001
Accuracy (95% CI)	0.88 (0.78-0.94)	0.82 (0.66-0.91)	>.99	0.77 (0.68-0.84)		0.80 (0.72-0.87)	.23	0.69 (0.61-0.76)		0.90 (0.83-0.94)	<.001

Abbreviations: AI, artificial intelligence; NPV, negative predictive value; PPV, positive predictive value

^a^ With AI: expert (n = 1).

A questionnaire was sent to the participants (eFigure 2 in Supplement 2). The joint-AI model’s mean (SD) effect on the endoscopist’s diagnosis was higher and was more preferred to use compared with the EUS-CNN model (3.46 [0.69] vs 2.54 [0.93]; *P* = .06) (eTable 9 in Supplement 2).

## Discussion

In this study, we developed a multimodal AI model (the joint-AI model), and a crossover trial was conducted on a prospective dataset to evaluate the ability of the model to assist endoscopists in diagnosing lesions in the pancreas. The strengths and potential paths for future clinical applications are discussed.

The joint-AI model possesses several advantages when compared with previously reported AI models.^12,13,14,15,16,17^ The first key advantage of the joint-AI model is its multimodal nature. In contrast to previous studies that built mainly single-modality models operating on EUS images only, the joint-AI model incorporated both EUS images and clinical information from multiple aspects. Throughout the training process, the interrelation between clinical and image features was continually refined, leading to a significant enhancement in performance.

The second advantage of the joint-AI model is the generalizability. The limitations of single-center designs in previous studies hindered necessary external validation, which is essential to evaluate the robustness of the model.^23^ To address this, 189 patients were collected across 3 independent centers to constitute the external validation sets. The joint-AI model maintained its robust performance on these external datasets. Furthermore, the model remained to be robust in a prospective dataset containing 130 patients from 2 hospitals, showcasing its ability to generalize to both time and space, which ensured broader applicability.

The third advantage of the joint-AI model lies in its alignment with clinical workflows. Existing AI models for classification of lesions in the pancreas predominantly focus on images from computed tomography scans or EUS images alone.^12,13,14,15,16,17,24^ However, in actual clinical practice, diagnoses often result from a comprehensive analysis of all the information available. Through emulating clinical decision-making processes by combining EUS images and clinical data, the joint-AI model has the potential to be integrated into clinical workflows with ease.

Considering our results, 2 major directions for clinical translation are recommended. First, the joint-AI model consistently demonstrated a robust level of sensitivity (0.88-0.99) and NPV (0.86-0.99) across diverse datasets. In contrast, EUS-FNA/B, the widely used diagnostic technique, merely reached a sensitivity of 0.85 to 0.89 and an NPV of 0.45 to 0.75.^7,8,9,10^ Although a positive pathologic diagnosis is not mandatory before surgery,^25^ considering the potential complications and the risk of resecting benign lesions inadvertently, acquiring a definitive diagnosis is optimal for patient care. Currently, ancillary techniques such as *KRAS* gene mutation analysis and repeat EUS-FNA/B are considered for cases with negative EUS-FNA/B results,^9^ subjecting patients to additional invasive procedures and economic burden. Therefore, the high sensitivity and NPV of the model may hold significant clinical implications by providing reliable complementary information to EUS-FNA/B. This information may eventually empower clinicians to make informed decisions in complex scenarios in which pathologic results are inconclusive.

Second, given the deep learning curve of the EUS examination and the lack of standardized and sufficient training procedures,^26,27^ the diagnostic ability varies greatly among endoscopists, particularly for less-experienced individuals. The interaction between endoscopists and the joint-AI model can potentially ameliorate this situation, as the performance of novice endoscopists was significantly improved with the AI assistance in the crossover trial. The transparency of the decision-making process in medical practice is highly valued. Still, deep learning models have been continually questioned for their black box nature, which may potentially hinder the clinical application.^28,29^ As reflected in the crossover trial, expert and senior endoscopists exhibited a greater tendency to reject the predictions of the AI models. To address this, interpretability analyses including Grad-CAM and SHAP were provided, and a lower rejection rate among expert and senior endoscopists was observed. For future clinical application, the results of interpretability analyses along with the predictions should be reported by the AI models simultaneously. Because clinicians can verify that the AI model is basing its predictions on the correct aspects of the EUS images and clinical features, they are more likely to accept the model’s prediction. In addition, rather than viewing the model as a black box, clinicians can engage with the model’s reasoning and use it as a complementary tool to support their decision-making process.

### Limitations

This study has some limitations. First, the crossover trial was conducted in silico instead of in a clinical environment. Only EUS images instead of videos were provided, and radiology findings were provided without the conclusion. The superior performance observed in our study cannot be directly applied to the actual clinical practice.^30^ Second, the prospective dataset is relatively small, comprising merely 130 patients, and only a crossover trial was conducted. In the future, we intend to design a multicenter randomized clinical trial with a larger and more diverse participant pool to further assess the clinical applicability of the joint-AI model. Third, despite our efforts in the interpretability analyses, there is still an urgent need for future research to enhance the transparency of multimodal AI models.

## Conclusions

In this randomized crossover trial of diagnosing solid lesions in the pancreas, the AI-assisted diagnostic process significantly improved the performance of novice endoscopists, while the interpretability analyses increased the acceptance of AI predictions by more experienced endoscopists. In the future, this joint-AI model, with its enhanced transparency in the decision-making process, has the potential to facilitate the diagnosis of solid lesions in the pancreas.

## Notes

Supplement 1.
Trial Protocol

Supplement 2.
eMethods.
**eTable 1.** Patient Demographics and Baseline Characteristics **eTable 2.** Performance of Model-1 in Internal and External Datasets **eTable 3.** Selection of Significant Clinical Features From Individual Categories **eTable 4.** Performance of Different Fusion Strategies in the Image Phase and Patient Phase **eTable 5.** Performance of Individual Endoscopists on the Prospective Dataset **eTable 6.** Performance of Model-1 and Endoscopists Without AI-Assistance on the Prospective Dataset **eTable 7.** Performance of Model-3 and Endoscopists Without AI-Assistance on the Prospective Dataset **eTable 8.** The Rate of Endoscopists Rejecting the AI-Assistance **eTable 9.** Comparison of the Impact Between EUS-CNN and Joint-AI on the Decision-Making of Endoscopists **eFigure 1.** Flow Diagram for Retrospective Data Collection **eFigure 2.** Questionnaire for Endoscopists on the Usage of the AI Models **eFigure 3.** ROC Analyses of Different Feature Fusion Strategies **eFigure 4.** AI Models’ Performance in Differentiating Carcinoma and Noncancerous Lesions in the Patient Phase **eFigure 5.** The Grad-CAM Analysis **eFigure 6.** The SHAP Analysis
eReferences.

Supplement 3.
Data Sharing Statement
