# Supplementary material for: Diagnosing Solid Lesions in the Pancreas With Multimodal Artificial Intelligence: A Randomized Crossover Trial
Source: JAMA Netw Open. 2024 Jul 19;7(7):e2422454. doi: 10.1001/jamanetworkopen.2024.22454 (PMC11259905; doi:10.1001/jamanetworkopen.2024.22454)
Supplement: Supplement 1. — Trial Protocol [file jamanetwopen-e2422454-s001.pdf]

Supplement 1. Trial protocol

Protocol Title: An artificial intelligence model in EUS for diagnosing pancreatic cancer and other solid lesions

IRB Number: TJ-IRB20220467

PI: Bin Cheng

Approval date: 29-Apr-2022

Table of Contents

1. Purpose **.......... 3**

2. Background **.......... 3**

3. The Retrospective Study Design **.......... 3**

3.1 Study design 3

3.2 Study population 4

3.3 Inclusion criteria 4

3.4 Exclusion criteria 4

3.5 Diagnostic criteria 4

4. The Retrospective Study Procedure **.......... 5**

4.1 Data collection and preprocessing 5

4.2 Model development 5

4.3 Data analysis 5

5. Outcomes of the Retrospective Study **.......... 6**

5.1 Primary outcomes 6

6. The Prospective Study Design **.......... 6**

6.1 Study design 6

6.2 Study population 6

6.3 Inclusion criteria 6

6.4 Exclusion criteria 7

6.5 Diagnostic criteria 7

7. The Prospective Study Procedure **.......... 7**

7.1 Screening 7

7.2 Recruitment 7

7.3 Patient data collection and preprocessing 8

7.4 Control group (Conventional diagnosis) 8

7.5 Intervention group (AI-assisted diagnosis) **9**
7.6 Data collection of the crossover study **9**
7.7 Data analysis **10**
8. Outcomes of the Prospective Study ***10***
8.1 Primary outcomes **10**
9. Statistical Analysis Plan ***11***
9.1 Power and sample size calculation **11**
9.2 Analysis of results **11**
10. Risks and Benefits ***11***
10.1 Risk to participants **11**
10.2 Benefit to participants **11**
11. Privacy and Confidentiality ***12***
12. References ***12***

1. Purpose

The purpose of this study is twofold: (1) To develop a multimodal AI model that integrates both clinical information and endoscopic ultrasound (EUS) images for the diagnosis of pancreatic solid lesions. The diagnostic performance of the model will be evaluated internally and externally. (2) To assess the AI model's potential to assist the diagnoses of endoscopists from varying levels of expertise in a prospective crossover trial.

We hope to find out whether the diagnostic performance of the multimodal AI model will improve compared to the model that relies solely on the EUS images. We hope that the improved diagnostic performance will allow the multimodal AI model to demonstrate robust performance across various external datasets collected at different institutions. Moreover, we hope that the multimodal AI model will improve the diagnostic performance of endoscopists in the crossover study. In the future, the findings of this study can serve as the first step towards incorporating this AI model into the clinical workflow of diagnosing pancreatic solid lesions.

2. Background

Pancreatic cancer is a prevalent cause of pancreatic masses with an overall 5-year survival rate of approximately 10%.[1] Accurate diagnosis of pancreatic solid lesions is crucial for appropriate patient management. EUS has emerged as a valuable technique for diagnosing pancreatic lesions, but its specificity in discriminating carcinoma from non-cancerous masses is suboptimal, ranging from 50% to 60%.[2] While artificial intelligence (AI) models such as convolutional neural network (CNN) have shown promise in differentiating pancreatic solid lesions in EUS images, prior studies predominantly lacked external validation and focused on a single modality.[3-5] Integrating multiple modalities, such as clinical information and EUS images, is expected to improve the robustness and accuracy of the diagnostic model and align better with real-world clinical practice, since in real-world medical context, the diagnosis is made after a comprehensive analysis of all the available clinical data.

3. The Retrospective Study Design

3.1 Study design

This is a retrospective, multi-center, diagnostic study.

3.2 Study population

We will include patients who underwent EUS procedures and received definite diagnosis of pancreatic solid lesions between January 2014 and December 2022 from four centers across China, including Wuhan Tongji Hospital (WHTJH), Nanjing Drum Tower Hospital (NJDTH), Peking Union Medical College Hospital (PUMCH), and Beijing Friendship Hospital (BJFH).

3.3 Inclusion criteria

1) Patients (aged ≥18 years) with pancreatic solid lesions
2) The carcinoma (CA) lesions: pancreatic ductal adenocarcinoma (PDAC), acinar cell carcinoma (ACC), and pancreatic squamous cell carcinoma (SCC).
3) The noncancerous lesions (Non-CA): pancreatic neuroendocrine tumor (pNET, Grade1 to Grade 3), solid pseudopapillary tumor (SPT), autoimmune pancreatitis (AIP), chronic pancreatitis (CP), and tuberculosis.
4) EUS equipment: EU-ME1 and EU-ME2 (Olympus Corporation, Tokyo, Japan) ultrasound systems equipped with either GF-UCT260 or GF-UCT240 curved linear echoendoscopes (Olympus Corporation, Tokyo, Japan).

3.4 Exclusion criteria

1) Aged <18 years
2) Received surgery of pancreas prior to the EUS procedure
3) Received chemotherapy, and radiotherapy because of pancreatic tumor prior to the EUS procedure.

3.5 Diagnostic criteria

1) PDAC, ACC, SCC, pNET, and SPT should be diagnosed pathologically by specimens obtained from EUS-FNA/B or surgery.
2) AIP should be diagnosed according to the International Consensus Diagnostic Criteria (ICDC) for AIP.
3) CP is diagnosed if there is neither malignancies detected in specimens acquired from EUS-FNA/B and/or surgery, nor a rapid progression of pancreatic diseases observed during the 6-

month follow-up period.

4) The diagnosis of tuberculosis will be based on a consensus reached through pathology, GeneXpert analysis, and the response to the anti-tuberculosis treatment.
5) Follow-up will be conducted for patients lack of following treatment and clinical outcomes.

4. The Retrospective Study Procedure

4.1 Data collection and preprocessing

1) Our team will collect the EUS images and clinical data, including personal history, clinical manifestations, medical history, laboratory tests, and radiology findings of the included patients.
2) The data collected at WHTJH will be used as training, validation, and testing datasets, while the data collected at NJDTH, PUMCH, and BJFH will be used as external testing datasets.
3) For EUS images, physician-captured still images and video-extracted images which can clearly present the pancreatic lesions will be selected. The preprocessing of the EUS images included removing the procedure-identifying information and poor-quality images resulting from the biopsy needle, annotation, and blurring.
4) According to the final diagnosis, the EUS images will be denoted as “0” for non-cancerous lesions and “1” for carcinoma lesions.

4.2 Model development

1) Model-1: This model is a CNN model trained with EUS images. The function of this model is to classify the pancreatic lesions into either CA or Non-CA according to the inputted EUS images.
2) Model-2: This model comprises multiple machine learning (ML) algorithms. This model will be trained with collected clinical information and will be used to select clinical features with critical diagnostic value.
3) Model-3: This model will combine the calculation results from the Model-1 and Model-2. Therefore, the function of Model-3 is to classify the pancreatic lesions into either CA or Non-CA based on EUS images and clinical features.

4.3 Data analysis

After the training of the AI models, the performance of the models will be evaluated in the internal testing and external testing datasets. The evaluation will be conducted in two phases: the image phase and the

patient phase.

In the image phase, Model-1 will give the prediction based on the individual image, while Model-3 will give the prediction based on the individual image and the corresponding clinical features.

In the patient phase, the predictions for each image of the patient (Model-1) or the predictions for each combination of image and clinical data of the patient (Model-3) will be aggregated to provide the final diagnosis of the given patient. The model's diagnostic performance will be assessed by comparing the predicted diagnosis with the actual classification (label) of the patient, and the performance metrics including accuracy, sensitivity, specificity, positive predictive value (PPV), negative predictive value (NPV), and area under the curve (AUC) will be calculated.

5. Outcomes of the Retrospective Study

5.1 Primary outcomes

The diagnostic performance of AI models:

1) The internal dataset and external datasets (NJDTH, PUMCH, BJFH): evaluated by metrics including accuracy, sensitivity, specificity, NPV, PPV, and AUC

6. The Prospective Study Design

6.1 Study design

This is a prospective, dual-center, randomized, open-label, crossover trial

6.2 Study population

After the completion of the model training, consecutive patients who underwent EUS examinations and received a definite diagnosis of pancreatic lesions will be prospectively enrolled from two centers (WHTJH, PUMCH).

6.3 Inclusion criteria

1) Patients (aged ≥18 years) with pancreatic solid lesions and scheduled to receive EUS procedure.
2) Informed consent obtained.
3) The carcinoma (CA) lesions: pancreatic ductal adenocarcinoma (PDAC), acinar cell carcinoma (ACC), and pancreatic squamous cell carcinoma (SCC).
4) The noncancerous lesions (Non-CA): pancreatic neuroendocrine tumor (pNET, Grade1 to Grade 3), solid pseudopapillary tumor (SPT), autoimmune pancreatitis (AIP), chronic pancreatitis (CP),

and tuberculosis.

6.4 Exclusion criteria

1) Aged <18 years
2) Received surgery of pancreas prior to the EUS procedure
3) Received chemotherapy, and radiotherapy because of pancreatic tumor prior to the EUS procedure.

6.5 Diagnostic criteria

1) PDAC, ACC, SCC, pNET, and SPT should be diagnosed pathologically by specimens obtained from EUS-FNA/B or surgery.
2) AIP should be diagnosed according to the International Consensus Diagnostic Criteria (ICDC) for AIP.
3) CP is diagnosed if there is neither malignancies detected in specimens acquired from EUS-FNA/B and/or surgery, nor a rapid progression of pancreatic diseases observed during the 6-month follow-up period.
4) The diagnosis of tuberculosis will be based on a consensus reached through pathology, GeneXpert analysis, and the response to the anti-tuberculosis treatment.
5) Follow-up will be conducted for patients lack of following treatment and clinical outcomes.

7. The Prospective Study Procedure

7.1 Screening

After completion of the model's training process, our team will screen for patients potentially meeting the inclusion criteria. Patients who are suspected of pancreatic solid lesions and scheduled to receive EUS procedures at WHTJH and PUMCH will be identified as potential candidates.

7.2 Recruitment

When eligible candidates are identified in the screening procedure, they will be approached by a member of our research team prior to their scheduled EUS procedure at WHTJH and PUMCH. The research team will explain the study objectives, procedures, and potential risks and benefits to the patients. Patients who

agree to participate in the study will be provided with an informed consent form to review. The research team will address any questions or concerns the patients may have about the study and the informed consent form. Patients who agree to participate in the study will be required to provide written informed consent. Upon obtaining the written consent, EUS images and clinical information of the patient who obtains a definite diagnosis will be collected and preprocessed as mentioned in the retrospective study procedure.

For the crossover study, endoscopists of varying level of expertise will be recruited for the study, including experts (who annually performed at least 300 EUS procedures with over ten years of experience), senior endoscopists (who annually performed at least 150 EUS procedures with over five years of experience), and novices (with over one year of experience in EUS). The recruited endoscopists will be provided with a detailed explanation of the study objectives, procedures, and their roles in the study. In addition, the function and the diagnostic performance of the AI models will be described.

According to the level of expertise, endoscopists will be randomly assigned in a 1:1 ratio to either the group that starts with AI assistance or the group that commences without AI assistance. After a washout period of at least two weeks, the endoscopists will switch groups and diagnose the same set of patients using the alternate approach (AI-assisted or conventional).

7.3 Patient data collection and preprocessing

As mentioned in the retrospective study procedure, the clinical information and EUS images will be collected from the patients who received definite diagnosis for their pancreatic solid lesions.

1) Clinical data, including personal history, clinical manifestations, medical history, laboratory tests, and radiology findings will be documented.
2) Physician-captured still images and video-extracted images that can clearly present the pancreatic lesions will be selected.
3) The preprocessing of the EUS images included removing the procedure-identifying information and poor-quality images resulted from biopsy needle, annotation and blurring.
4) None of the endoscopists in the crossover study will participate in this process, and they will all be masked to the personal information, EUS reports, pathological results, and clinical diagnosis of the involved patients.

7.4 Control group (Conventional diagnosis)

Endoscopists in the control group will only be provided with EUS images and clinical information. Two

diagnoses are required to be made. 1) Diagnosis-1: Endoscopists are required to make this diagnosis according to the EUS images only. 2) Diagnosis-2: Endoscopists are required to make this diagnosis based on both EUS images and clinical information.

Endoscopists will be required to classify each pancreatic lesion as either cancerous (CA) or non-cancerous (Non-CA). While the primary requirement is to provide this binary classification, endoscopists will have the option to offer a more specific diagnosis (e.g., PDAC, pNET, AIP, CP, SPT, etc.) if they feel confident in doing so.

Endoscopists will document their diagnoses using a standardized online form. The form will include fields for the binary CA/Non-CA classification, as well as an optional field for the specific diagnosis.

7.5 Intervention group (AI-assisted diagnosis)

Endoscopists in the intervention group will have access to the predictions of AI models when making their first diagnoses. Next, interpretability analyses, including gradient-weighted class activation mapping (Grad-CAM) and Shapley additive explanations (SHAP) algorithms will be provided to endoscopists. With the predictions given by the AI models and the interpretability analyses, endoscopists will be required to make the second diagnosis on the same set of patients.

For each patient, the endoscopists will be provided with the EUS images and clinical information alongside the given predictions. They are only required to classify the pancreatic lesion into either CA or Non-CA, while they can give the specific classification. Endoscopists will document their diagnoses in a standardized online form which will include fields for the binary CA/Non-CA classification, as well as an optional field for the specific diagnosis.

7.6 Data collection of the crossover study

The diagnoses made by endoscopists will be collected from the standardized online forms which the endoscopists finished in both the intervention and the control group.

At the end of the crossover study, a questionnaire will be sent to the endoscopists, asking the impact of the AI models on their decision-making process and their preference to the AI models.

7.7 Data analysis

We will analyze the diagnostic performance of each endoscopist with or without the AI assistance. The diagnostic performance of AI models in the prospective dataset will be analyzed. We will compare the diagnostic performance of AI models with endoscopists from different level of expertise. The rate of endoscopist from different level of expertise rejecting the AI-assistance will be analyzed. The impact of the AI models on the decision-making process and the endoscopists' preference for the AI models will be analyzed. The rejection rate of the endoscopists with or without the interpretability analyses will be compared.

8. Outcomes of the Prospective Study

8.1 Primary outcomes

The diagnostic performance of endoscopists with or without the AI-assistance

1) Measured by metrics including sensitivity, specificity, accuracy, positive predictive value (PPV), negative predictive value (NPV), and area under the curve (AUC)

8.2 Secondary outcomes

1) The diagnostic performance of AI models in the prospective dataset
    a. Measured by metrics including sensitivity, specificity, accuracy, positive predictive value (PPV), negative predictive value (NPV), and area under the curve (AUC)
2) The impact of AI models on the decision-making process of endoscopists
    a. The impact will be scored by endoscopists in the questionnaire at the end of the study
3) Endoscopists' preference for AI models
    a. This will be scored by endoscopists in the questionnaire at the end of the study
4) The rejection rate of the expert and senior endoscopists with or without the interpretability analysis
    a. The total rejection rate (TRR) is defined as the proportion of cases in which expert and senior endoscopists disagree with the predictions of AI models. It will be calculated as follows:

       TRR = (Number of cases where endoscopists disagree with the the predictions of AI models) / (Total number of cases)
    b. The false rejection rate (FRR) is defined as the proportion of cases in which expert and senior endoscopists incorrectly reject the predictions of AI models. It will be calculated as follows:

FRR = (Number of cases where endoscopists incorrectly reject the predictions of AI models) / (Total number of cases where endoscopists disagree with the predictions of AI models)

9. Statistical Analysis Plan

9.1 Power and sample size calculation

The sample size is calculated based on the primary hypothesis which is the diagnostic performance of novices improved significantly with AI assistance. We anticipate that the diagnostic accuracy of the AI model in the prospective dataset to be 88% and the accuracy of novices is 72% based on previous results. The estimated sample size was 126 with a type 1 error rate of 0.05 and power of 0.90. We slightly enlarged the sample size to 150.

9.2 Analysis of results

The primary outcome, the diagnostic performance of endoscopists with or without the AI-assistance will be evaluated by metrics including accuracy, sensitivity, specificity, positive predictive value (PPV), negative predictive value (NPV), and area under the curve (AUC). The McNemar test will be used to compare the accuracy, sensitivity, and specificity. Generalized score statistics will be utilized to compare the PPV and NPV. The optimal cutoff value of the receiver operating characteristic (ROC) curve is determined when the Youden Index is maximized. A Wilcoxon matched-pairs signed rank test will be used to compare the impact of models on the diagnosis of endoscopists. Chi-square analysis will be used to compare the rejection rate of endoscopists with or without interpretability analyses and endoscopists' preferences for the AI models. A two-sided p-value less than 0.05 is considered statistically significant.

10. Risks and Benefits

10.1 Risk to participants

This study poses negligible risk to its participants, with only EUS images and clinical information collected. In addition, during the data collection process, patient's personal information will not be collected and the data will be securely stored within our research team.

10.2 Benefit to participants

There is no direct benefit to its participants. However, in the future, we hope the findings of this study can serve as the first step towards incorporating this AI model into the clinical workflow, improving the

clinical management for the patients with pancreatic solid lesions.

11. Privacy and Confidentiality

Only EUS images and clinical information will be collected from the patients and personal information will not be collected. Data will be securely stored within our research team.

During the crossover study, endoscopists will only receive the EUS images and clinical information of the patient without any personal information or procedure identifying information.

12. References

1. Cai J, Chen H, Lu M, et al. Advances in the epidemiology of pancreatic cancer: Trends, risk factors, screening, and prognosis. *Cancer Lett*. Nov 1 2021;520:1-11. doi:10.1016/j.canlet.2021.06.027
2. Giovannini M. The place of endoscopic ultrasound in bilio-pancreatic pathology. *Gastroentérologie Clinique et Biologique*. 2010;34(8-9):436-445. doi:10.1016/j.gcb.2010.05.004
3. Marya NB, Powers PD, Chari ST, et al. Utilisation of artificial intelligence for the development of an EUS-convolutional neural network model trained to enhance the diagnosis of autoimmune pancreatitis. *Gut*. Jul 2021;70(7):1335-1344. doi:10.1136/gutjnl-2020-322821
4. Tonozuka R, Itoi T, Nagata N, et al. Deep learning analysis for the detection of pancreatic cancer on endosonographic images: a pilot study. *J Hepatobiliary Pancreat Sci*. Jan 2021;28(1):95-104. doi:10.1002/jhbp.825
5. Udriștoiu AL, Cazacu IM, Gruionu LG, et al. Real-time computer-aided diagnosis of focal pancreatic masses from endoscopic ultrasound imaging based on a hybrid convolutional and long short-term memory neural network model. *PLoS One*. 2021;16(6):e0251701. doi:10.1371/journal.pone.0251701
