# Supplementary material for: Diagnosing Solid Lesions in the Pancreas With Multimodal Artificial Intelligence: A Randomized Crossover Trial
Source: JAMA Netw Open. 2024 Jul 19;7(7):e2422454. doi: 10.1001/jamanetworkopen.2024.22454 (PMC11259905; doi:10.1001/jamanetworkopen.2024.22454)
Supplement: Supplement 2. — eMethods. eTable 1. Patient Demographics and Baseline Characteristics eTable 2. Performance of Model-1 in Internal and External Datasets eTable 3. Selection of Significant Clinical Features From Individual Categories eTable 4. Performance of Different Fusion Strategies in the Image Phase and Patient Phase eTable 5. Performance of Individual Endoscopists on the Prospective Dataset eTable 6. Performance of Model-1 and Endoscopists Without AI-Assistance on the Prospective Dataset eTable 7. Performance of Model-3 and Endoscopists Without AI-Assistance on the Prospective Dataset eTable 8. The Rate of Endoscopists Rejecting the AI-Assistance eTable 9. Comparison of the Impact Between EUS-CNN and Joint-AI on the Decision-Making of Endoscopists eFigure 1. Flow Diagram for Retrospective Data Collection eFigure 2. Questionnaire for Endoscopists on the Usage of the AI Models eFigure 3. ROC Analyses of Different Feature Fusion Strategies eFigure 4. AI Models’ Performance in Differentiating Carcinoma and Noncancerous Lesions in the Patient Phase eFigure 5. The Grad-CAM Analysis eFigure 6. The SHAP Analysis eReferences. [file jamanetwopen-e2422454-s002.pdf]

Supplementary Online Content

Cui H, Zhao Y, Xiong S, et al. Diagnosing solid lesions in the pancreas with multimodal artificial intelligence: a randomized crossover trial. *JAMA Netw Open.* 2024;7(7):e2422454. doi:10.1001/jamanetworkopen.2024.22454

eMethods.

eTable 1. Patient Demographics and Baseline Characteristics

eTable 2. Performance of Model-1 in Internal and External Datasets

eTable 3. Selection of Significant Clinical Features From Individual Categories

eTable 4. Performance of Different Fusion Strategies in the Image Phase and Patient Phase

eTable 5. Performance of Individual Endoscopists on the Prospective Dataset

eTable 6. Performance of Model-1 and Endoscopists Without AI-Assistance on the Prospective Dataset

eTable 7. Performance of Model-3 and Endoscopists Without AI-Assistance on the Prospective Dataset

eTable 8. The Rate of Endoscopists Rejecting the AI-Assistance

eTable 9. Comparison of the Impact Between EUS-CNN and Joint-AI on the Decision-Making of Endoscopists

eFigure 1. Flow Diagram for Retrospective Data Collection

eFigure 2. Questionnaire for Endoscopists on the Usage of the AI Models

eFigure 3. ROC Analyses of Different Feature Fusion Strategies

eFigure 4. AI Models' Performance in Differentiating Carcinoma and Noncancerous Lesions in the Patient Phase

eFigure 5. The Grad-CAM Analysis

eFigure 6. The SHAP Analysis

eReferences.

This supplementary material has been provided by the authors to give readers additional information about their work.

© 2024 Cui H et al. *JAMA Network Open.*

eMethods

Data collection and preprocessing

1. **Clinical information.** Clinical information including personal history, clinical manifestations, medical history, laboratory tests, and radiology findings was documented from electronic medical records. For missing values, the data entry for that variable was left blank.
    - **1.1.** Personal history: sex, age, BMI, history of smoking, history of alcohol consumption.
    - **1.2.** Clinical symptoms: abdominal pain, weight loss, jaundice, diarrhea, vomiting, back pain, symptoms of hypoglycemia, weight gain.
    - **1.3.** Medical history: new-onset diabetes within 2 years, tumor history in other systems, chronic pancreatitis, long-term diabetes, hepatitis B virus, and hypertension. As some diabetic medications have been reported to be associated with risk for pancreatic cancer,[1,2] we also documented the medications being used at the time of the EUS procedure, including metformin, sulfonylureas, thiazolidinediones, and insulin.
    - **1.4.** Laboratory tests: direct bilirubin, CA19-9, CEA, amylase, lipase.
    - **1.5.** Radiology findings: appearance of the lesion including CT attenuation in the pancreatic parenchymal phase, MRI T1-weighted signal, MRI T2-weighted signal, and diffusion-weighted signal (DWI), presence of pancreatic duct dilation, presence of common bile duct dilation, presence of pancreatic enlargement, presence of pancreatic parenchymal atrophy.
2. **EUS images.**
    - **2.1.** EUS procedures were conducted using EU-ME1 and EU-ME2 (Olympus Corporation, Tokyo, Japan) ultrasound systems equipped with either GF-UCT260 or GF-UCT240 curved linear echoendoscopes (Olympus Corporation, Tokyo, Japan).
    - **2.2.** All still images captured by endoscopists during the procedure and images extracted from the video clips of the recorded video which could clearly present the pancreatic lesion, were extracted and organized into corresponding patient folders within each cohort.
    - **2.3.** In cases where the patients underwent multiple EUS examinations, images and videos were stored in separate case folders.
    - **2.4.** Every image was labeled as either “1” for carcinoma lesions or “0” for other lesions according to the final diagnosis of the corresponding patient.
3. **Image preprocessing procedures.**
    - **3.1.** The preprocessing of the EUS images included removing the procedure-identifying information and poor-quality images that might sabotage the efficacy and reliability of the developed AI models.
    - **3.2.** Details such as patient names, admission numbers, procedure locations or timestamps, and the white-light picture located at the lower right corner of the EUS image were removed by cropping.
    - **3.3.** Poor-quality images, potentially resulting from air, blurring, the presence of biopsy needles, elastography, or other artifacts not intrinsic to the original EUS images, were eliminated to prevent potential biases in the model.
    - **3.4.** Image sizes: We resized all images to a uniform dimension of 224 × 224 pixels. This image size was chosen to ensure compatibility with the pre-trained ResNet50 model architecture.
    - **3.5.** Image channel: The EUS images were converted from the RGB color space to the BGR color space to match the expected input format of the pre-trained CNN model.

Model training and testing

1. **Datasets distribution.** The collected datasets were split according to institutions. The dataset from WHTJH was randomly partitioned into training, validation, and internal testing with a ratio of 6:2:2. For the development of Model-1, the split was performed at the individual image level, addressing the imbalanced quantity of images between patients. Furthermore, to mitigate the potential risk of overestimating the model’s performance due to closely correlated images extracted from videos being allocated into both training and testing subsets, the video-extracted images were carefully reviewed and similar images were removed. The datasets sourced from the other three institutions were exclusively designated as external testing datasets. This segregation of the datasets allowed for an objective evaluation of the generalizability of the trained models.
2. **Model-1.** This EUS image-based convolution neural network model was developed using a ResNet50 CNN implemented in TensorFlow 2.3.0. Transfer learning was employed, incorporating pre-trained weights from ImageNet. The final classification layer was removed. The top eight layers from the pre-trained model were unfrozen for fine-tuning the parameters using our training and validation dataset. The output of the base model was passed through a global average pooling layer to reduce the spatial dimensions of the feature maps. The resulting features were then flattened and passed through a dense layer with a sigmoid activation function to

© 2024 Cui H et al. *JAMA Network Open.*

produce the probability (a continuous variable from zero to one) of the input image. The final output of the Model-1 was binary, being either carcinoma (CA) or non-cancerous (non-CA). To prevent overfitting, three methods including data augmentation, dropout, and early stopping were adopted.

- **2.1.** Firstly, data augmentation was utilized. The augmentation techniques included random horizontal and vertical translations (range ratios of 20% and 30%, respectively), shear transformation (stretching images along the horizontal or vertical direction to a degree of 20 degrees), rotation (up to 10 degrees), zoom (maximum range of 30%), constant filling of empty regions after transformations, and random vertical and horizontal flipping.
- **2.2.** Secondly, a dropout layer was applied to the GAP layer with a rate of 0.50.
- **2.3.** Thirdly, early stopping was implemented to halt training when validation loss failed to decrease for ten consecutive epochs. Model-1 was configured to trained for a maximum of 200 epochs. While the actual number of training epochs for Model-1 was 64, due to the early stopping criteria.
- **2.4.** Grid search was used to find the optimal hyperparameters of the model. Specifically, the learning rate was chosen among 0.1, 0.01, and 0.001. The batch size was chosen among 16, 32, and 64. The dropout rate was chosen among 0.2, 0.3, 0.4, and 0.5. After conducting the grid search and evaluating the model's performance for each combination of hyperparameters, the learning rate was set to 0.001, the batch size was set to 16, and the dropout rate was set to 0.5.
- **2.5.** The model was trained by the Adam optimizer with an initial learning rate of 0.001, a batch size of 16, weight decay of 0.000001, and momentum of 0.90.

3. Model-2 (Selection of significant clinical features). Feature selection was conducted to lower the risk of overfitting and reduce computation burden.[3]

- **3.1.** The training dataset used for the ML models was the same as Model-1, consisting of the clinical data collected from 351 patients at our center (WHTJH).
- **3.2.** Firstly, a total of 36 clinical features (sex, age, BMI, history of smoking, history of alcohol consumption, abdominal pain, weight loss, jaundice, diarrhea, vomiting, back pain, symptoms of hypoglycemia, weight gain, new-onset diabetes within 2 years, tumor history in other systems, chronic pancreatitis, long term diabetes, hepatitis B virus, hypertension, metformin, sulfonylureas, thiazolidinediones, insulin, direct bilirubin, CA19-9, CEA, amylase, lipase, appearance of the lesion including CT attenuation in the pancreatic parenchymal phase, MRI T1-weighted signal, MRI T2-weighted signal, DWI, presence of pancreatic duct dilation, presence of common bile duct dilation, presence of pancreatic enlargement, presence of pancreatic parenchymal atrophy) were categorized into five groups according to their nature: personal history, medical history, clinical symptoms, laboratory test and radiology findings.
- **3.3.** Next, features from the same category were arranged into various combinations to train several machine learning (ML) models. Given the differences in data types and characteristics among the five categories of clinical features, it was reasonable to expect that the optimal machine learning algorithm for capturing the relevant patterns and relationships within each category might differ. Therefore, multiple ML algorithms were employed during the training process, including Gaussian naive Bayes (GNB), k-nearest neighbors (KNN), logistic regression (LR), random forest (RF), decision tree (DT), support vector machine (SVM), and gradient boosting decision tree (GBDT). The probabilities of the patients having CA were produced based on the inputted clinical features, and the final output of the ML models was binary (CA or Non-CA).
- **3.4.** The optimal combinations of ML algorithm and features for each category were determined based on the diagnostic accuracy evaluated by clinical data of the 88 patients from the internal test dataset.

4. Model-3 (the joint-AI model).

- **4.1.** The inputs to Model-3 included outputs from the linear layers of Model-1 and selected clinical features from Model-2. Because of the multilayer nature of the CNN network, the features extracted by layers closer to the output are more abstract.[4] Therefore, three fusion strategies were used to generate the input vector for Model-3.
  - 4.1.1. Strategy A fused the penultimate layer of Model-1 (including 2048 image features) with clinical features selected by Model-2 (including 24 clinical features).
  - 4.1.2. Strategy B fused the last layer of Model-1 (including 1 feature) with the probabilities calculated by Model-2 (including 5 features).
  - 4.1.3. Strategy C directly fused the outputs from Model-1 (including 1 feature) and Model-2 (including 5 features).
- **4.2.** The Model-3 was a multi-layer perceptron model with two fully-connected layers implemented in TensorFlow 2.3.0. The model's input layer had 64 nodes, followed by an additional 32 nodes. Two dropout layers, with a rate of 0.5, were applied to mitigate overfitting. The output layer was connected to a

© 2024 Cui H et al. *JAMA Network Open.*

sigmoid activation function to produce the probability of the input image. The final output of the Model-3 was binary (CA or Non-CA).

4.3. Similarly, grid search was used to find the optimal hyperparameters of the model. The learning rate was set to 0.001, the batch size was set to 16, and the dropout rate was set to 0.5.

4.4. This model was optimized by Adam with a learning rate set to 0.001,[5] and binary cross entropy was used as the loss function. To prevent overfitting, early stopping was implemented to halt training if the validation loss failed to decrease for ten consecutive epochs. Model-3 was configured to trained for a maximum of 40 epochs. While the actual number of training epochs for Model-3 was 24, due to the early stopping criteria.

4.5. The diagnostic efficacy of models built using the three fusion strategies was evaluated. The model with the best performance was further evaluated in a prospective dataset.

5. **Interpretability analysis.** Firstly, gradient-weighted class activation mapping (Grad-CAM) was applied to Model-1.[6] The heatmap generated by Grad-CAM indicated the regions within the EUS images that significantly influenced the predictions. On the other hand, shapley additive explanations (SHAP) was implemented to analyze the output of the Model-3.[7] This approach provided both localized explanations, tailored to specific patients, and global explanations considering all instances of the model. Through SHAP, the contributions of individual elements in the prediction process were quantitatively indicated.[8,9]

© 2024 Cui H et al. *JAMA Network Open.*

**eTable 1.** Patient Demographics and Baseline Characteristics					
**Characteristic**	**Datasets**				***P* value** [b]
	**Training & Validation dataset, N = 351** [a]	**Internal test dataset, N = 88** [a]	**External test datasets, N = 189** [a]	**Prospective test dataset, N = 130** [a]	
**Diseases**					.01
CA	213 (61%)	57 (65%)	93 (49%)	84 (65%)	
AIP	40 (11%)	12 (14%)	26 (14%)	14 (11%)	
CP	48 (14%)	7 (8%)	21 (11%)	24 (18%)	
NET (G1-G3)	27 (8%)	9 (10%)	27 (14%)	7 (5%)	
SPT	12 (3%)	2 (2%)	18 (10%)	0 (0%)	
TB	4 (1%)	0 (0%)	4 (2%)	1 (1%)	
Others [c]	7 (2%)	1 (1%)	0 (0%)	0 (0%)	
**Gender**					.82
Male	229 (65%)	54 (61%)	117 (62%)	81 (62%)	
Female	122 (35%)	34 (39%)	72 (38%)	49 (38%)	
**Age**					.19
Median (IQR)	58 (50, 65)	60 (54, 65)	59 (50, 68)	60 (52, 67)	
**BMI**					.58
Median (IQR)	21.0 (19.0, 23.0)	22.0 (19.0, 23.0)	23.0 (19.0, 24.0)	21.0 (19.0, 24.0)	
**Smoking history**					.004
Yes	75 (22%)	19 (22%)	33 (38%)	23 (18%)	
No	271 (78%)	68 (78%)	54 (62%)	106 (82%)	
**Alcohol consumption history**					<.001
Yes	64 (18%)	14 (16%)	31 (36%)	14 (11%)	
No	282 (82%)	73 (84%)	56 (64%)	114 (89%)	
**Abdominal pain**					.002
Yes	254 (72%)	56 (64%)	45 (52%)	90 (69%)	
No	97 (28%)	32 (36%)	42 (48%)	40 (31%)	
**Weight loss**					<.001
Yes	137 (39%)	34 (39%)	50 (57%)	33 (25%)	
No	214 (61%)	54 (61%)	37 (43%)	97 (75%)	
**Jaundice**					.008
Yes	77 (22%)	16 (18%)	33 (38%)	30 (23%)	
No	274 (78%)	72 (82%)	54 (62%)	100 (77%)	
**Diarrhea**					.007
Yes	14 (4%)	6 (7%)	10 (11%)	2 (2%)	
No	337 (96%)	82 (93%)	77 (89%)	128 (98%)	
**Vomit**					<.001
Yes	25 (7%)	5 (6%)	21 (24%)	16 (12%)	
No	326 (93%)	83 (94%)	66 (76%)	114 (88%)	
**Back pain**					.03
Yes	42 (12%)	11 (13%)	21 (24%)	20 (15%)	
No	309 (88%)	77 (88%)	66 (76%)	110 (85%)	
**Hypoglycemia**					.12
Yes	6 (2%)	4 (5%)	0 (0%)	1 (1%)	
No	345 (98%)	84 (95%)	87 (100%)	129 (99%)	
**Weight gain**					.76
Yes	2 (1%)	1 (1%)	1 (1%)	1 (1%)	
No	349 (99%)	87 (99%)	86 (99%)	129 (99%)	

© 2024 Cui H et al. *JAMA Network Open.*

**eTable 1.** Patient Demographics and Baseline Characteristics					
**Characteristic**	**Datasets**				***P* value** [b]
	**Training & Validation dataset, N = 351** [a]	**Internal test dataset, N = 88** [a]	**External test datasets, N = 189** [a]	**Prospective test dataset, N = 130** [a]	
**New onset diabetes**					.03
Yes	51 (15%)	9 (10%)	17 (20%)	9 (7%)	
No	300 (85%)	79 (90%)	70 (80%)	121 (93%)	
**Other system tumor history**					.06
Yes	13 (4%)	2 (2%)	9 (10%)	5 (4%)	
No	338 (96%)	86 (98%)	78 (90%)	125 (96%)	
**Chronic pancreatitis**					.52
Yes	25 (7%)	3 (3%)	4 (5%)	10 (8%)	
No	326 (93%)	85 (97%)	83 (95%)	120 (92%)	
**Long term diabetes**					.04
Yes	21 (6%)	9 (10%)	13 (15%)	14 (11%)	
No	330 (94%)	79 (90%)	74 (85%)	116 (89%)	
**HBV**					.38
Yes	11 (3%)	3 (3%)	0 (0%)	4 (3%)	
No	340 (97%)	85 (97%)	87 (100%)	126 (97%)	
**Metformin**					<.001
Yes	7 (2%)	2 (2%)	9 (10%)	0 (0%)	
No	344 (98%)	86 (98%)	78 (90%)	130 (100%)	
**Sulfonylureas**					.47
Yes	9 (3%)	1 (1%)	4 (5%)	5 (4%)	
No	342 (97%)	87 (99%)	83 (95%)	125 (96%)	
**Thiazolidinediones**					.009
Yes	17 (5%)	8 (9%)	13 (15%)	8 (6%)	
No	334 (95%)	80 (91%)	74 (85%)	122 (94%)	
**Insulin**					<.001
Yes	28 (8%)	3 (3%)	17 (20%)	4 (3%)	
No	323 (92%)	85 (97%)	70 (80%)	126 (97%)	
**Direct bilirubin**					.12
Median (IQR)	4 (3, 10)	5 (3, 8)	4 (2, 18)	5 (3, 17)	
**CA19-9**					.06
Median (IQR)	59 (13, 491)	67 (16, 479)	32 (8, 330)	74 (16, 616)	
**CEA**					.002
Median (IQR)	3 (2, 5)	3 (2, 5)	2 (1, 4)	3 (2, 8)	
**Blood sugar**					<.001
Median (IQR)	6.02 (5.19, 7.75)	6.09 (5.35, 7.71)	5.50 (4.86, 6.77)	6.20 (5.47, 7.78)	
**Amylase**					<.001
Median (IQR)	28 (18, 69)	33 (21, 93)	61 (43, 95)	44 (25, 86)	
**Lipase**					.07
Median (IQR)	56 (32, 150)	70 (27, 132)	89 (46, 220)	75 (36, 238)	
**Location**					.12
Head	226 (64%)	62 (70%)	118 (62%)	71 (55%)	
Body	71 (20%)	15 (17%)	49 (26%)	28 (22%)	
Tail	32 (9%)	8 (9%)	14 (7%)	19 (15%)	
Body, tail	0 (0%)	0 (0%)	0 (0%)	1 (1%)	
Diffuse	22 (6%)	3 (3%)	8 (4%)	11 (8%)	

© 2024 Cui H et al. *JAMA Network Open.*

eTable 1. Patient Demographics and Baseline Characteristics

Characteristic	Datasets				*P* value [b]
	Training & Validation dataset, N = 351 [a]	Internal test dataset, N = 88 [a]	External test datasets, N = 189 [a]	Prospective test dataset, N = 130 [a]	
**Size (mm)**					.03
Median (IQR)	33 (25, 41)	32 (22, 38)	30 (23, 37)	32 (27, 40)	
**CT attenuation**					<.001
Hypoattenuating	173 (74%)	39 (67%)	96 (79%)	71 (81%)	
Isoattenuating	42 (18%)	17 (29%)	7 (6%)	11 (13%)	
Hyperattenuating	18 (8%)	2 (3%)	19 (16%)	6 (7%)	
**Pancreatic parenchyma enlargement**					.005
Yes	53 (16%)	11 (13%)	23 (14%)	28 (29%)	
No	285 (84%)	72 (87%)	145 (86%)	68 (71%)	
**Pancreatic parenchyma atrophy**					<.001
Yes	43 (12%)	14 (16%)	50 (30%)	18 (19%)	
No	308 (88%)	74 (84%)	118 (70%)	75 (81%)	
**MRI T1-weighted**					.30
Hypointensity	125 (87%)	30 (83%)	63 (90%)	34 (92%)	
Isointensity	12 (8%)	6 (17%)	3 (4%)	2 (5%)	
Hyperintensity	4 (3%)	0 (0%)	4 (6%)	0 (0%)	
Mixed Intensity	2 (1%)	0 (0%)	0 (0%)	1 (3%)	
**MRI T2-weighted**					.46
Hypointensity	6 (3%)	5 (10%)	5 (6%)	3 (5%)	
Isointensity	11 (6%)	4 (8%)	2 (2%)	3 (5%)	
Hyperintensity	154 (86%)	39 (80%)	74 (90%)	48 (87%)	
Mixed Intensity	8 (4%)	1 (2%)	1 (1%)	1 (2%)	
**DWI signal intensity**					.01
Hyperintensity	145 (88%)	41 (100%)	71 (97%)	43 (93%)	
Normal	20 (12%)	0 (0%)	2 (3%)	3 (7%)	
**Common bile duct dilation**					.26
Yes	102 (38%)	29 (42%)	74 (48%)	52 (42%)	
No	164 (62%)	40 (58%)	79 (52%)	71 (58%)	
**Pancreatic duct dilation**					.76
Yes	173 (57%)	47 (62%)	89 (60%)	69 (63%)	
No	128 (43%)	29 (38%)	59 (40%)	41 (37%)	

a. n (%)
b. Pearson's Chi-squared test; Kruskal-Wallis rank sum test; Fisher's exact test
c. Others: lymphoma (n=4), perivascular epithelioid cell tumor (n=1), Spindle cell tumor (n=1), accessory spleen (n=1), High-grade sarcoma (n=1)

© 2024 Cui H et al. *JAMA Network Open.*

eTable 2. Performance of Model-1 in Internal and External Datasets

Datasets	Sensitivity (95%CI)	Specificity (95%CI)	PPV (95%CI)	NPV (95%CI)	Accuracy (95%CI)
**Image phase:**					
Internal testing	0.92 (0.90-0.94)	0.92 (0.90-0.94)	0.91 (0.88-0.93)	0.93 (0.91-0.94)	0.92 (0.90-0.93)
NJDTH	0.93 (0.90-0.95)	0.67 (0.63-0.71)	0.63 (0.59-0.67)	0.94 (0.91-0.96)	0.77 (0.74-0.80)
PUMCH	0.81 (0.72-0.88)	0.65 (0.58-0.72)	0.55 (0.47-0.63)	0.87 (0.80-0.91)	0.70 (0.65-0.76)
BJFH	0.80 (0.66-0.90)	0.76 (0.58-0.88)	0.82 (0.68-0.91)	0.73 (0.56-0.86)	0.78 (0.67-0.86)
**Patient phase:**					
Internal testing	0.88 (0.83-0.91)	0.75 (0.69-0.81)	0.85 (0.80-0.89)	0.79 (0.72-0.85)	0.83 (0.79-0.86)
NJDTH	0.84 (0.72-0.92)	0.76 (0.63-0.86)	0.78 (0.66-0.87)	0.83 (0.70-0.91)	0.80 (0.72-0.87)
PUMCH	0.63 (0.44-0.78)	0.76 (0.60-0.87)	0.65 (0.46-0.80)	0.74 (0.58-0.85)	0.70 (0.58-0.80)
BJFH	0.80 (0.55-0.93)	0.75 (0.41-0.93)	0.85 (0.60-0.96)	0.67 (0.35-0.88)	0.78 (0.58-0.90)

WHTJH, Wuhan Tongji Hospital; NJDTH, Nanjing Drum Tower Hospital; PUMCH, Peking Union Medical College Hospital; BJFH, Beijing Friendship Hospital

© 2024 Cui H et al. *JAMA Network Open.*

eTable 3. Selection of Significant Clinical Features From Individual Categories

Categories	Selected features [a]	ML algorithms	Accuracy [b]
Personal history	sex, age, BMI, smoking history, alcohol consumption history	RF	80.81%
Medical history	new onset diabetes, HBV, sulfonylureas	DT	66.00%
Clinical symptoms	abdominal pain, weight loss, jaundice, diarrhea, vomit	SVM	67.43%
Laboratory tests	direct bilirubin, CA19-9, CEA, blood sugar	GBDT	88.07%
Radiology findings	CT attenuation, MRI T1-weighted signal, pancreatic enlargement, pancreatic parenchymal atrophy, DWI signal intensity, common bile duct dilation, pancreatic duct dilation	RF	85.21%

RF: random forest, DT: decision tree, SVM: support vector machine, GBDT: gradient boosted decision tree.

a. A total of 24 features selected by the respective ML algorithms from the original 36 features.

b. The set of the selected features demonstrated the highest accuracy within each category.

© 2024 Cui H et al. *JAMA Network Open.*

eTable 4. Performance of Different Fusion Strategies in the Image Phase and Patient Phase

Strategies	Sensitivity (95%CI)	Specificity (95%CI)	PPV (95%CI)	NPV (95%CI)	Accuracy (95%CI)
**Image phase**					
Strategy A	0.96 (0.95-0.97)	0.92 (0.90-0.94)	0.94 (0.92-0.95)	0.95 (0.93-0.96)	0.94 (0.93-0.95)
Strategy B	0.99 (0.98-0.99)	0.98 (0.96-0.98)	0.98 (0.97-0.99)	0.99 (0.98-0.99)	0.98 (0.98-0.99)
**Patient phase**					
Strategy A	0.96 (0.92-0.97)	0.94 (0.89-0.97)	0.96 (0.93-0.98)	0.92 (0.88-0.96)	0.95 (0.92-0.97)
Strategy B	0.99 (0.97-1.00)	0.98 (0.94-0.99)	0.98 (0.96-0.99)	0.98 (0.95-0.99)	0.98 (0.97-0.99)
Strategy C	0.98 (0.95-0.99)	0.92 (0.87-0.95)	0.95 (0.92-0.97)	0.96 (0.92-0.98)	0.96 (0.93-0.97)

© 2024 Cui H et al. *JAMA Network Open.*

eTable 5. Performance of Individual Endoscopists on the Prospective Dataset

Endoscopists	Sensitivity (95%CI)	Specificity (95%CI)	PPV (95%CI)	NPV (95%CI)	Accuracy (95%CI)
**Without clinical information:**					
Expert 1	0.75 (0.53-0.89)	0.69 (0.43-0.87)	0.79 (0.57-0.91)	0.64 (0.39-0.84)	0.73 (0.56-0.85)
Expert 2	0.73 (0.52-0.87)	0.82 (0.52-0.95)	0.89 (0.67-0.97)	0.6 (0.36-0.80)	0.76 (0.59-0.87)
Senior 1	0.56 (0.34-0.75)	0.67 (0.39-0.86)	0.71 (0.45-0.88)	0.50 (0.28-0.72)	0.60 (0.42-0.75)
Senior 2	0.59 (0.36-0.78)	1.00 (0.74-1.00)	1.00 (0.72-1.00)	0.61 (0.39-0.80)	0.75 (0.57-0.87)
Senior 3	0.78 (0.52-0.92)	0.56 (0.27-0.81)	0.73 (0.48-0.89)	0.62 (0.30-0.86)	0.70 (0.49-0.84)
Senior 4	0.60 (0.39-0.78)	0.67 (0.39-0.86)	0.75 (0.50-0.90)	0.50 (0.28-0.72)	0.62 (0.45-0.77)
Novice 1	0.84 (0.62-0.94)	0.46 (0.23-0.71)	0.70 (0.49-0.84)	0.67 (0.35-0.88)	0.69 (0.51-0.82)
Novice 2	0.54 (0.29-0.77)	0.38 (0.14-0.69)	0.58 (0.32-0.81)	0.33 (0.12-0.64)	0.48 (0.28-0.68)
Novice 3	0.47 (0.26-0.69)	0.91 (0.62-0.98)	0.89 (0.56-0.98)	0.53 (0.32-0.73)	0.64 (0.46-0.79)
Novice 4	0.70 (0.47-0.87)	0.73 (0.43-0.90)	0.80 (0.55-0.93)	0.62 (0.36-0.82)	0.71 (0.53-0.85)
Novice 5	0.40 (0.20-0.64)	0.75 (0.41-0.93)	0.75 (0.41-0.93)	0.4 (0.20-0.64)	0.52 (0.33-0.71)
Novice 6	0.33 (0.15-0.58)	0.75 (0.41-0.93)	0.71 (0.36-0.92)	0.38 (0.18-0.61)	0.48 (0.29-0.67)
**With clinical information:**					
Expert 1	0.85 (0.64-0.95)	0.85 (0.58-0.96)	0.89 (0.69-0.97)	0.78 (0.52-0.92)	0.85 (0.69-0.93)
Expert 2	0.91 (0.72-0.97)	0.91 (0.62-0.98)	0.95 (0.77-0.99)	0.83 (0.55-0.95)	0.91 (0.76-0.97)
Senior 1	0.67 (0.44-0.84)	0.83 (0.55-0.95)	0.86 (0.60-0.96)	0.62 (0.39-0.82)	0.73 (0.56-0.86)
Senior 2	0.59 (0.36-0.78)	1.00 (0.74-1.00)	1.00 (0.72-1.00)	0.61 (0.39-0.80)	0.75 (0.57-0.87)
Senior 3	0.86 (0.60-0.96)	0.56 (0.27-0.81)	0.75 (0.50-0.90)	0.71 (0.36-0.92)	0.74 (0.53-0.87)
Senior 4	0.80 (0.58-0.92)	0.92 (0.65-0.98)	0.94 (0.73-0.99)	0.73 (0.48-0.89)	0.84 (0.68-0.93)
Novice 1	0.63 (0.41-0.81)	0.85 (0.58-0.96)	0.86 (0.60-0.96)	0.61 (0.39-0.80)	0.72 (0.55-0.84)
Novice 2	0.77 (0.50-0.92)	0.75 (0.41-0.93)	0.83 (0.55-0.95)	0.67 (0.35-0.88)	0.76 (0.55-0.89)
Novice 3	0.35 (0.17-0.59)	0.91 (0.62-0.98)	0.86 (0.49-0.97)	0.48 (0.28-0.68)	0.57 (0.39-0.73)
Novice 4	0.76 (0.53-0.90)	0.73 (0.43-0.90)	0.81 (0.57-0.93)	0.67 (0.39-0.86)	0.75 (0.57-0.87)

© 2024 Cui H et al. *JAMA Network Open.*

Endoscopists	Sensitivity (95%CI)	Specificity (95%CI)	PPV (95%CI)	NPV (95%CI)	Accuracy (95%CI)
Novice 5	0.80 (0.55-0.93)	0.75 (0.41-0.93)	0.86 (0.60-0.96)	0.67 (0.35-0.88)	0.78 (0.58-0.90)
Novice 6	0.40 (0.20-0.64)	0.88 (0.53-0.98)	0.86 (0.49-0.97)	0.44 (0.23-0.67)	0.56 (0.37-0.74)
**With AI-assistance:**					
Expert 1	0.80 (0.58-0.92)	0.85 (0.58-0.96)	0.89 (0.67-0.97)	0.73 (0.48-0.89)	0.82 (0.66-0.91)
Senior 1	0.72 (0.49-0.88)	0.83 (0.55-0.95)	0.87 (0.62-0.96)	0.67 (0.42-0.85)	0.77 (0.59-0.88)
Senior 2	0.53 (0.31-0.74)	1.00 (0.74-1.00)	1.00 (0.70-1.00)	0.58 (0.36-0.77)	0.71 (0.53-0.85)
Senior 3	1.00 (0.78-1.00)	0.56 (0.27-0.81)	0.78 (0.55-0.91)	1.00 (0.56-1.00)	0.83 (0.63-0.93)
Senior 4	0.90 (0.70-0.97)	0.92 (0.65-0.98)	0.95 (0.75-0.99)	0.85 (0.58-0.96)	0.91 (0.76-0.97)
Novice 1	0.89 (0.69-0.97)	0.92 (0.67-0.99)	0.94 (0.74-0.99)	0.86 (0.60-0.96)	0.91 (0.76-0.97)
Novice 2	0.92 (0.67-0.99)	0.75 (0.41-0.93)	0.86 (0.60-0.96)	0.86 (0.49-0.97)	0.86 (0.65-0.95)
Novice 3	0.94 (0.73-0.99)	0.91 (0.62-0.98)	0.94 (0.73-0.99)	0.91 (0.62-0.98)	0.93 (0.77-0.98)
Novice 4	0.94 (0.73-0.99)	0.91 (0.62-0.98)	0.94 (0.73-0.99)	0.91 (0.62-0.98)	0.93 (0.77-0.98)
Novice 5	0.87 (0.62-0.96)	0.88 (0.53-0.98)	0.93 (0.68-0.99)	0.78 (0.45-0.94)	0.87 (0.68-0.95)
Novice 6	0.87 (0.62-0.96)	0.88 (0.53-0.98)	0.93 (0.68-0.99)	0.78 (0.45-0.94)	0.87 (0.68-0.95)

© 2024 Cui H et al. *JAMA Network Open.*

eTable 6. Performance of Model-1 and Endoscopists Without AI-Assistance on the Prospective Dataset

Metrics	Model-1	Experts (n=2)		Seniors (n=4)		Novices (n=6)	
		Metrics	*P value*	Metrics	*P value*	Metrics	*P value*
**Sensitivity (95%CI)**	0.93 (0.85-0.97)	0.74 (0.59-0.85)	.02	0.62 (0.50-0.73)	< .001	0.56 (0.46-0.66)	< .001
**Specificity (95%CI)**	0.74 (0.60-0.84)	0.75 (0.55-0.88)	1.00	0.73 (0.58-0.84)	1.00	0.66 (0.53-0.77)	.40
**PPV (95%CI)**	0.87 (0.78-0.92)	0.84 (0.69-0.92)	.87	0.78 (0.66-0.87)	.44	0.73 (0.62-0.82)	.04
**NPV (95%CI)**	0.85 (0.71-0.93)	0.62 (0.44-0.77)	.03	0.55 (0.42-0.67)	< .001	0.48 (0.38-0.59)	< .001
**Accuracy (95%CI)**	0.86 (0.79-0.91)	0.74 (0.62-0.83)	.13	0.66 (0.57-0.74)	< .001	0.60 (0.52-0.67)	< .001

© 2024 Cui H et al. *JAMA Network Open.*

eTable 7. Performance of Model-3 and Endoscopists Without AI-Assistance on the Prospective Dataset

**Metrics**	**Model-3**	**Experts (n=2)**		**Seniors (n=4)**		**Novices (n=6)**	
		**Metrics**	***P value***	**Metrics**	***P value***	**Metrics**	***P value***
**Sensitivity (95%CI)**	0.92 (0.84-0.96)	0.88 (0.75-0.95)	1.00	0.72 (0.61-0.82)	.002	0.61 (0.51-0.70)	< .001
**Specificity (95%CI)**	0.93 (0.82-0.98)	0.88 (0.69-0.96)	1.00	0.84 (0.71-0.92)	.34	0.81 (0.70-0.89)	.06
**PPV (95%CI)**	0.96 (0.90-0.99)	0.92 (0.80-0.97)	.62	0.88 (0.77-0.94)	.12	0.84 (0.74-0.91)	.004
**NPV (95%CI)**	0.86 (0.74-0.93)	0.80 (0.62-0.91)	.48	0.66 (0.53-0.77)	.01	0.56 (0.46-0.66)	< .001
**Accuracy (95%CI)**	0.92 (0.86-0.96)	0.88 (0.78-0.94)	.55	0.77 (0.68-0.84)	.001	0.69 (0.61-0.76)	< .001

© 2024 Cui H et al. *JAMA Network Open.*

eTable 8. The Rate of Endoscopists Rejecting the AI-Assistance

Endoscopists	Total rejection rate [a]	False rejection rate [b]	Odds ratio (95% CI) [c]	*P* value
**Without interpretability analysis**				
Expert & senior endoscopists	19.86%	75.86%	2.15 (1.12-4.16)	.02
Novices	10.32%	68.75%		
**With interpretability analysis**				
Expert & senior endoscopists	9.32%	45.45%	0.71 (0.32-1.58)	.40

a. Total rejection rate: (number of cases endoscopists disagree with prediction of the joint-AI) / (total number of cases)
b. False rejection rate: (number of cases endoscopists falsely reject the prediction of the joint-AI) / (total number of cases endoscopists disagree with the prediction of the joint-AI)
c. Odds ratio was calculated by the total rejection rate between novices and expert & senior endoscopists with or without the interpretability analysis

© 2024 Cui H et al. *JAMA Network Open.*

eTable 9. Comparison of the Impact Between EUS-CNN and Joint-AI on the Decision-Making of Endoscopists [a]

Questionnaire	EUS-CNN	Joint-AI	*P* value
The impact of the AI model on the diagnoses made by endoscopists [b]	2.54 (0.93)	3.46 (0.69)	.06
Number of endoscopists who preferred to use this model	2	9	.009

a. During the crossover study, to help endoscopists better understand the nature of each AI model, Model-1 was named as "EUS-CNN" and Model-3 was named as "joint-AI"
b. Data denoted by mean (SD)

© 2024 Cui H et al. *JAMA Network Open.*

eFigure 1. Flow Diagram for Retrospective Data Collection

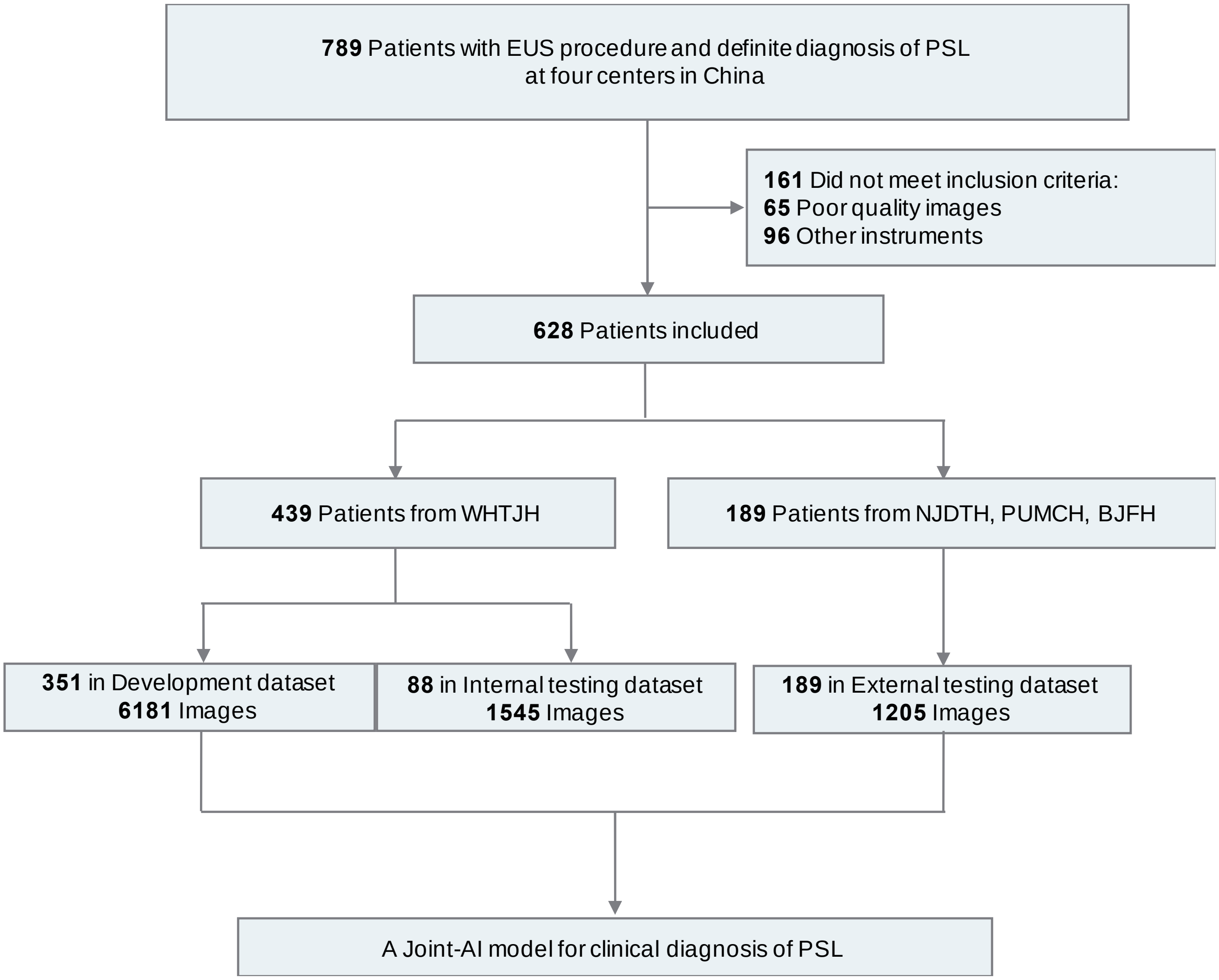

The datasets for training and validation were retrospectively collected**.** PSL, pancreatic solid lesions. NJDTH, Nanjing Drum Tower Hospital; PUMCH, Peking Union Medical College Hospital; BJFH, Beijing Friendship Hospital.

© 2024 Cui H et al. *JAMA Network Open.*

eFigure 2. Questionnaire for Endoscopists on the Usage of the AI Models

Questionnaire - AI models feedback

Please finish the following questions accordingly.

***01.To what extent does the EUS-CNN model influence your decision?**

○ 1（Never） ○ 2 ○ 3 ○ 4 ○ 5（Always）

***02.To what extent does the joint-CNN model influence your decision?**

○ 1 (Never) ○ 2 ○ 3 ○ 4 ○ 5 (Always)

***03.Suppose in the real-world medical practice, which model do you prefer to assist your diagnosis?**

○ EUS-AI ○ Joint-AI ○ Never

Endoscopists were required to finish the questionnaire at the end of the study. The “joint-CNN” was the previous name of the “joint-AI” model. To avoid potential confusion, we changed to name to the “joint-AI” model when drafting this paper. The sample is the translated version, as the original one is written in Chinese.

© 2024 Cui H et al. *JAMA Network Open.*

eFigure 3. ROC Analyses of Different Feature Fusion Strategies

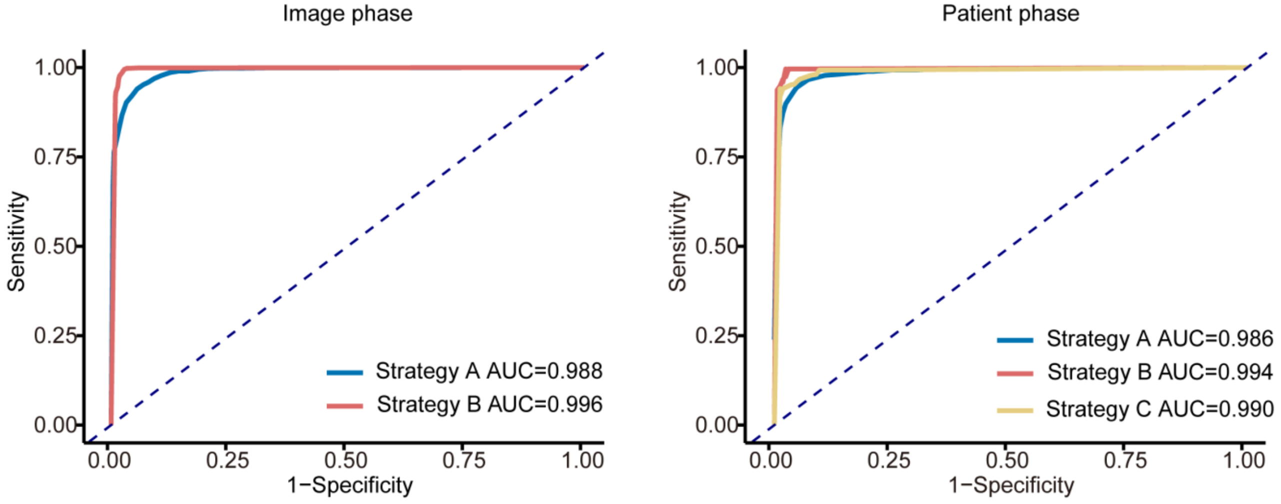

The models developed by strategy A, B and C were compared on the internal testing dataset in image and patient phase. Strategy C, due to its direct fusion of predictions according to entire images and clinical features of patients, could only be evaluated in the patient phase. The strategy with the best performance (strategy B) was selected to develop the final joint-AI model. ROC, receiver operating characteristic; AUC, area under the curve.

© 2024 Cui H et al. *JAMA Network Open.*

eFigure 4. AI Models' Performance in Differentiating Carcinoma and Noncancerous Lesions in the Patient Phase

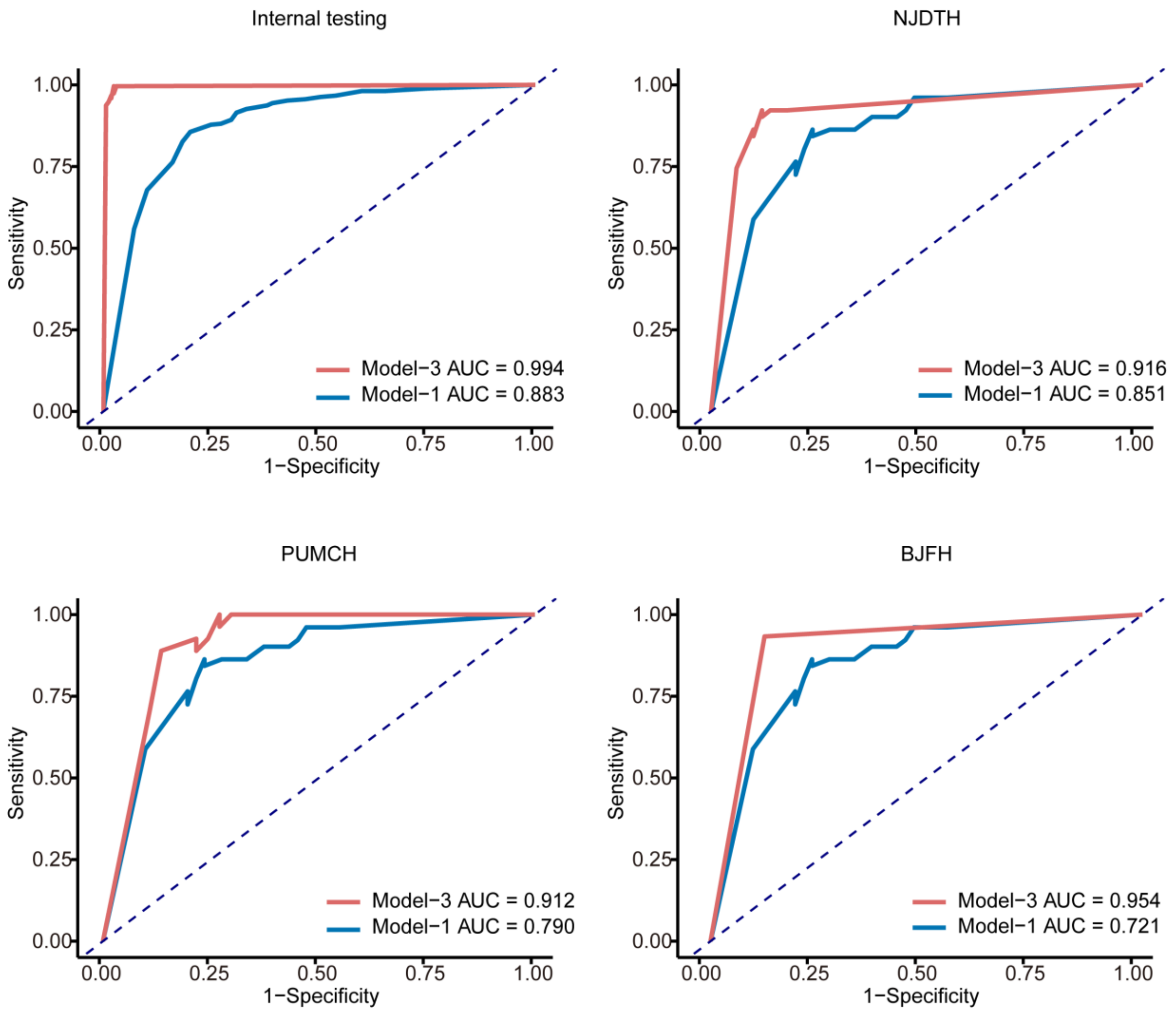

The performance of the AI models in the patient phase. Model-3 was developed based on both clinical information and EUS images, whereas Model-1 was trained on EUS images only. The internal testing dataset was collected from WHTJH, Wuhan Tongji Hospital. Three external testing datasets were involved: NJDTH, Nanjing Drum Tower Hospital; PUMCH, Peking Union Medical College Hospital; BJFH, Beijing Friendship Hospital. ROC, receiver operating characteristic; AUC, area under the curve.

© 2024 Cui H et al. *JAMA Network Open.*

eFigure 5. The Grad-CAM Analysis

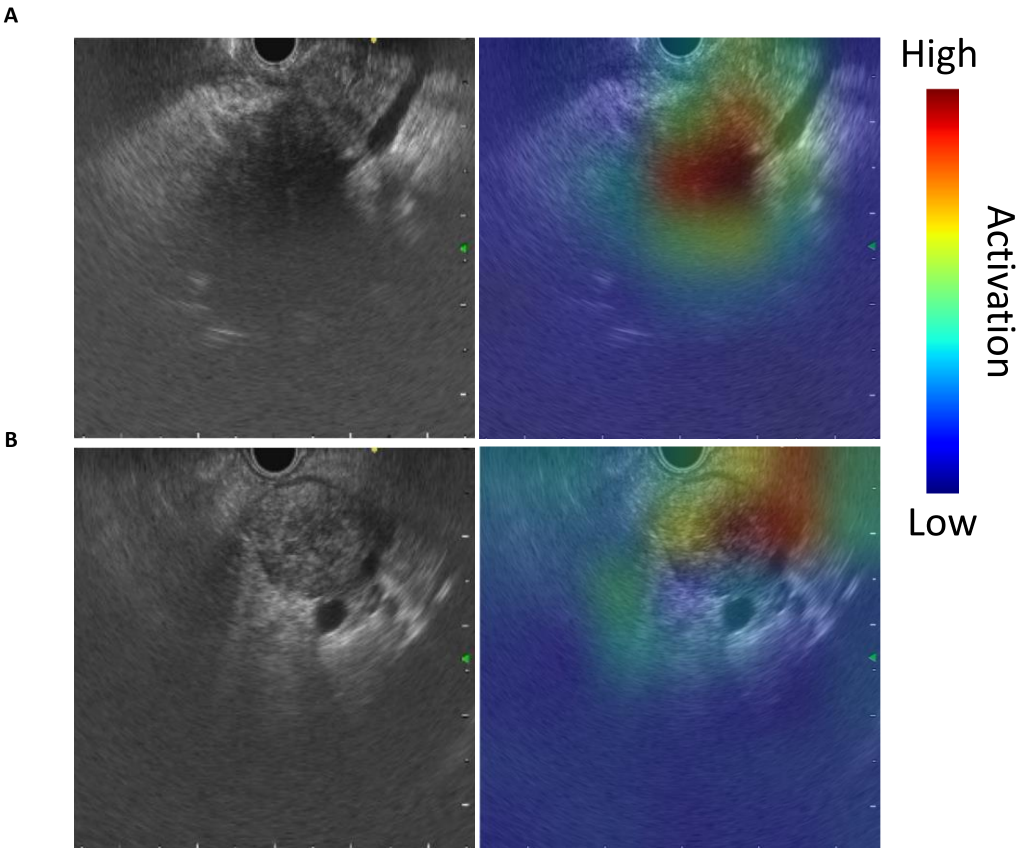

Representative EUS images and their corresponding Grad-CAM heatmaps. The heatmaps display the model's focused area within the EUS images. The upper pair presents a carcinoma lesion (A), while the lower pair exhibits a benign lesion resulting from chronic pancreatitis (B). The presence of a heated area in the Grad-CAM heatmap for the chronic pancreatitis can be attributed to its shared image features with the pancreatic cancer. However, despite the presence of these shared features, the model's predicted probability for the image of chronic pancreatitis does not exceed the diagnostic threshold for carcinoma, leading to a negative prediction. Grad-CAM, gradient-weighted class activation mapping.

© 2024 Cui H et al. *JAMA Network Open.*

eFigure 6. The SHAP Analysis

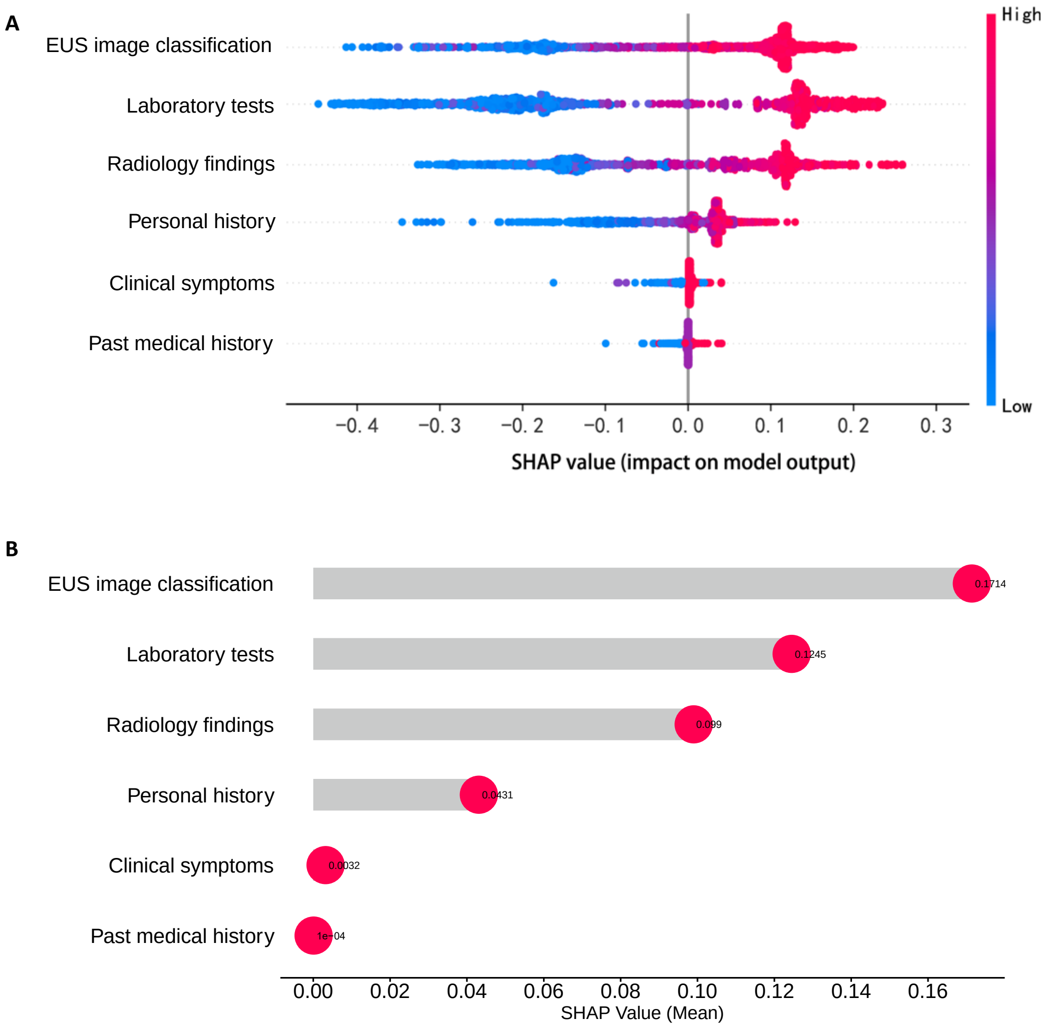

The upper one depicts the global explanation provided by the SHAP analysis, illustrating the impact of features from different categories on the output of the joint-AI model. This analysis encompasses all cases in the joint-AI model, with each dot representing a specific case (A). The lower one is a representative local explanation generated by the SHAP algorithm for a specific patient (B). SHAP, Shapley additive explanations.

© 2024 Cui H et al. *JAMA Network Open.*

eReferences.

1. Bodmer M, Becker C, Meier C, Jick SS, Meier CR. Use of antidiabetic agents and the risk of pancreatic cancer: a case-control analysis. *Am J Gastroenterol*. Apr 2012;107(4):620-6. doi:10.1038/ajg.2011.483
2. Bosetti C, Rosato V, Li D, et al. Diabetes, antidiabetic medications, and pancreatic cancer risk: an analysis from the International Pancreatic Cancer Case-Control Consortium. *Ann Oncol*. Oct 2014;25(10):2065-2072. doi:10.1093/annonc/mdu276
3. van Timmeren JE, Cester D, Tanadini-Lang S, Alkadhi H, Baessler B. Radiomics in medical imaging-"how-to" guide and critical reflection. *Insights Imaging*. Aug 12 2020;11(1):91. doi:10.1186/s13244-020-00887-2
4. Baltrusaitis T, Ahuja C, Morency LP. Multimodal Machine Learning: A Survey and Taxonomy. *IEEE Trans Pattern Anal Mach Intell*. Feb 2019;41(2):423-443. doi:10.1109/TPAMI.2018.2798607
5. Takase T, Oyama S, Kurihara M. Effective neural network training with adaptive learning rate based on training loss. *Neural Netw*. May 2018;101:68-78. doi:10.1016/j.neunet.2018.01.016
6. Selvaraju RR, Cogswell M, Das A, Vedantam R, Parikh D, Batra D. Grad-cam: Visual explanations from deep net- works via gradient-based localization, Venice, Italy, 22–29 October 2017. *In Proceedings of the IEEE International Conference on Computer Vision*. 2017:618-626.
7. Lundberg SM, Lee S-I. A unified approach to interpreting model predictions. presented at: Proceedings of the 31st International Conference on Neural Information Processing Systems; 2017; Long Beach, California, USA.
8. Antoniadi AM, Du Y, Guendouz Y, et al. Current Challenges and Future Opportunities for XAI in Machine Learning-Based Clinical Decision Support Systems: A Systematic Review. *Applied Sciences*. 2021;11(11)doi:10.3390/app11115088
9. Price WN. Big data and black-box medical algorithms. *Sci Transl Med*. Dec 12 2018;10(471)doi:10.1126/scitranslmed.aao5333

© 2024 Cui H et al. *JAMA Network Open.*
