# Supplementary material for: Diagnosing Solid Lesions in the Pancreas With Multimodal Artificial Intelligence: A Randomized Crossover Trial
Source: JAMA Netw Open. 2024 Jul 19;7(7):e2422454. doi: 10.1001/jamanetworkopen.2024.22454 (PMC11259905; doi:10.1001/jamanetworkopen.2024.22454)
Supplement: Supplement 3. — Data Sharing Statement [file jamanetwopen-e2422454-s003.pdf]

Data Sharing Statement

Cui. Diagnosing Solid Lesions in the Pancreas With Multimodal Artificial Intelligence. *JAMA Netw Open*. Published July 19, 2024. doi:10.1001/jamanetworkopen.2024.22454

Data
Data available: Yes
Data types: Deidentified participant data
How to access data: b.cheng@tjh.tjmu.edu.cn.
When available: With publication

Supporting Documents
Document types: None

Additional Information
Who can access the data: Researchers whose proposed use of the data has been approved
Types of analyses: For a specified purpose
Mechanisms of data availability: with a signed data access agreement
